# Inference about quantitative traits under selection: a Bayesian revisitation for the post-genomic era

**DOI:** 10.1186/s12711-022-00765-z

**Published:** 2022-12-02

**Authors:** Daniel Gianola, Rohan L. Fernando, Chris C. Schön

**Affiliations:** 1 Department of Animal and Dairy Sciences, University of Wisconsin, Madison, WI USA; 2 Department of Animal Science, Iowa State University, Ames, IA USA; 3 Department of Plant Breeding, Technical University of Munich, Freising, Germany

## Abstract

**Background:**

Selection schemes distort inference when estimating differences between treatments or genetic associations between traits, and may degrade prediction of outcomes, e.g., the expected performance of the progeny of an individual with a certain genotype. If input and output measurements are not collected on random samples, inferences and predictions must be biased to some degree. Our paper revisits inference in quantitative genetics when using samples stemming from some selection process. The approach used integrates the classical notion of fitness with that of missing data. Treatment is fully Bayesian, with inference and prediction dealt with, in an unified manner. While focus is on animal and plant breeding, concepts apply to natural selection as well. Examples based on real data and stylized models illustrate how selection can be accounted for in four different situations, and sometimes without success.

**Results:**

Our flexible “soft selection” setting helps to diagnose the extent to which selection can be ignored. The clear connection between probability of missingness and the concept of fitness in stylized selection scenarios is highlighted. It is not realistic to assume that a fixed selection threshold *t* holds in conceptual replication, as the chance of selection depends on observed and unobserved data, and on unequal amounts of information over individuals, aspects that a “soft” selection representation addresses explicitly. There does not seem to be a general prescription to accommodate potential distortions due to selection. In structures that combine cross-sectional, longitudinal and multi-trait data such as in animal breeding, balance is the exception rather than the rule. The Bayesian approach provides an integrated answer to inference, prediction and model choice under selection that goes beyond the likelihood-based approach, where breeding values are inferred indirectly.

**Conclusions:**

The approach used here for inference and prediction under selection may or may not yield the best possible answers. One may believe that selection has been accounted for diligently, but the central problem of whether statistical inferences are good or bad does not have an unambiguous solution. On the other hand, the quality of predictions can be gauged empirically via appropriate training-testing of competing methods.

## Background

Quantitative genetics explains and describes inheritance of complex traits such as many diseases in humans and animals or agriculturally relevant targets, e.g., yield and product quality in maize or dairy cattle. It focuses on statistical quantities, e.g., allelic and haplotype frequencies, locus effect sizes, means, variances and covariances between individuals or traits. Theory-derived parameters like heritability and genetic correlations and linkage disequilibrium are of interest as well. Although they are interpretable, these parameters may not provide a meaningful mechanistic explanation of genetic systems and represent abstractions. Interaction is key in biological processes, both at the biochemical (e.g., cycles) and population levels, e.g., the shifting balance theory of evolution [1, 2]. Yet, quantitative genetics heavily relies on the additive genetic model, both in its pre- and post-genomics versions. Actually, this model is a crucial tool in the armamentarium of animal and plant breeders and also plays a role in the “polygenic scores” used for prediction in human medicine [3]. Hence, learning well the parameters of additive models is important.

All unknown quantities are inferred using finite samples of experimental or observational data. Quantitative genetic models have been informed by phenotypes and genealogies [4–7] and more recently by molecular markers [8–11]. Also, it is increasingly feasible to obtain (often expensively) joint measures of the genome, epigenome, metabolome, proteome, metagenome, behavior, robustness, resilience and sustainability from samples of individuals. Such inputs are fed to prediction and decision machines used for artificial selection, field fertilization, animal management and disease treatment algorithms. However, data are often not representative of a target population because of selection schemes in animals and plants, and expensive measurements are seldom taken at random. Likewise, in medical trials, individuals not meeting certain criteria or “culling levels” are excluded and there may be a non-random dropout of accepted patients, i.e., some abandon the study due to treatment effects. Selection schemes distort inference when estimating differences between treatments or genetic associations between traits, or degrade prediction of outcomes, e.g., the expected performance of the progeny of an individual with a certain genotype. If input and output measurements are not taken on random samples, inferences and predictions may be biased to some extent.

The preceding problem is not novel in quantitative genetics. Ideally, properties of estimators and predictors should be studied relative to a setting representing the selection or dropout process occurring, which is not an easy task. There are many potential scenarios: selection may be by truncation of a distribution, it may be disruptive with two tails of the distribution selected, or aimed at stabilizing a population near some optimum [12]. However, there are situations in which it is impossible or awkward to model the selection process in a simple manner. For instance, if individuals are heterogeneous, related through complex pedigree loops or possess unequal amounts of information, classical balanced-data selection index formulae [13–15] or stylized treatments for parameter estimation are not entirely adequate.

With the advent of genomics and “big” post-genomic data, distortions produced by selection may have been exacerbated. Best linear unbiased prediction (BLUP) evolved into GBLUP, with “G” denoting “genomic” and Bayesian linear regression models emerged as competing prediction machines [10, 16–20]. Although the data used in genome-based models is rarely random, the “selection problem” has been seldom discussed in depth from a theoretical perspective. Earlier studies [21–23] pointed out that if a process (e.g., selection) that leads to “missing data” depends on observed data only and on parameters that are “separate” from those of the statistical model employed for analysis, selection is said to be ignorable. However, when data is available on “survivors” only, the selection process and its parameters must be considered in the analysis for appropriate inference or prediction. In between, there is a plethora of scenarios.

This paper revisits inference of quantitative genetic unknowns when using samples stemming from a selection process. Our approach integrates classical notions of fitness with that of missing data. The treatment is fully Bayesian, with inference and prediction dealt with, in an unified manner. The focus is on animal and plant breeding but concepts apply to natural selection as well.

## Bayesian setting

Let ${\mathbf {y}}$ and ${\theta}$ be vectors of observable variables and unknown quantities, respectively. In genetic contexts, ${\theta}$ may include genomic and epigenomic site effects, genetic and environmental components of (co) variance, nuisance location parameters, latent quantities such as breeding values and yet to be observed (future) phenotypes. One seeks to learn ${\theta}$ from the observed ${\mathbf {y}}.$ In a Bayesian treatment all elements of ${\theta}$ are viewed as randomly varying, reflecting aleatory or causal uncertainty [24] and are assigned some prior distribution with density $p({\theta} {|}Hyp,M)$, where *Hyp* denotes known hyper-parameters under some model *M*, e.g., a linear regression with a certain linear structure and distributional assumptions; model $M^{\prime },$ say, may assume a thick-tailed residual distribution, while *M* may have a non-linear part and treat the residuals as Gaussian.

Without natural or artificial selection, the joint density of ${\mathbf {y}}$ and ${\theta}$ under model *M* is:

1 $$p({\mathbf {y}},{\theta} {|}Hyp,M)=p({\mathbf {y}}|{\theta}, M)p({\theta} {|}Hyp,M),$$

where $p({\mathbf {y}}|{\theta}{,}M)$ is the density of the data-generating distribution under *M*, with ${\theta}$ fixed. The posterior density of ${\theta}$ is

2 $$p({\theta} {|} {\mathbf{y}},Hyp,M)=\frac{p({\mathbf {y}}|{\theta}, M)p({\theta} {|}Hyp,M)}{\int p({\mathbf {y}}|{\theta}, M)p({\theta} {|}Hyp,M)d{\theta}}=\frac{p({\mathbf {y}}|{\theta},M)p({\theta} {|}Hyp,M)}{p({\mathbf {y}}{|}Hyp,M)}.$$

The denominator $p({\mathbf {y}}{|}Hyp,M)$ is the Bayesian marginal density of the data, that is, the reciprocal of the integration constant of the posterior density under *M*. The latter depends on ${\mathbf {y}}$ and on *M*, but not on ${\theta}{,}$ which has been averaged out using *p*$({\theta}{{|}}Hyp,M)$ as weight function [25]. Often, the target of the analysis is a component of ${\theta}$, e.g., the vector of breeding values. Partitioning the entire parameter vector into ${\theta}_{i}$ and ${\theta}_{-i},$ where ${\theta}_{-i}$ is ${\theta}$ without ${\theta}_{i}$, the marginal posterior density of sub-vector ${\theta}_{i}$ is $p({\theta}_{i}{|}{\mathbf {y}},Hyp,M)=\int p({\theta}{|}{\mathbf{y}},Hyp,M)d{\theta}_{-i}$ [25]. Bayesian computations are typically done via Monte Carlo sampling procedures.

## Fitness depending on observed data

Various formulations of fitness functions appear in [12, 26–29]. Suppose natural or artificial selection operate on phenotypes through a fitness function $H(\mathbf {y|}{\varphi}{,}{\theta}),$ where ${\varphi}$ is a parameter vector that may be distinct from ${\theta}$ and that does not enter into the process generating observed data ${\mathbf {y}}$. The fitness function may depend on phenotypes linearly or non-linearly and is proportional to the probability that an individual possessing some observed attributes will reproduce or survive.

Let $p({\theta}{,} {\varphi}{|}Hyp,M)$ be the joint prior density of all parameters. The post-selection joint density of ${\mathbf {y}}$, ${\theta}$ and ${\varphi}$ is:

3 $$\begin{aligned} p_{s}({\mathbf {y}},{{\theta},{\varphi}{|}}Hyp,M)&=\frac{H( {\mathbf {y}}{|}{\varphi},{\theta})p({\mathbf {y}}|{\theta},M)p( {\theta},{\varphi}{|}Hyp,M)}{ { \iiint } H({\mathbf {y}}{|}{\varphi},{\theta})p({\mathbf {y}}|{\theta},M)p( {{\theta},{\varphi}{|}}Hyp,M)d{\mathbf {y}}d{\theta}d{\varphi}} \\&=\frac{H({\mathbf {y}}{|}{\varphi},{\theta})p({\mathbf {y}}| {\theta},M)p({\theta},{\varphi}{{|}}Hyp,M)}{ { \iiint } H({\mathbf {y}}|{\varphi},{\theta})p({\mathbf {y}},{\varphi},{\theta} {|}Hyp,M)d{\mathbf {y}} d{\theta}d{\varphi}} \\&=\frac{H({\mathbf {y}}{|}{\varphi},{\theta})p({\mathbf {y}}|{\theta},M)p({\theta ,\varphi |}Hyp,M)}{\overline{H}} \end{aligned}$$

Here, $\overline{H}$ is “Bayesian mean fitness”; it does not involve ${\mathbf {y}}$, ${\theta}$ and ${\varphi}$ since these vectors have been averaged out, so $\overline{H}^{-1}$ acts as an integration constant. Note that $\dfrac{H({\mathbf {y}}{|}{\varphi},{\theta})}{\overline{H}}$ is the relative fitness associated with ${\mathbf {y}}$ at fixed ${\varphi}{,}{\theta};$ values conferring lower fitness are assigned less density following selection. Without selection, $H({\mathbf {y}}{|} {\varphi}{,}{\theta })$ is constant and $H(\mathbf {y}|{\varphi}{,}{\theta})=\overline{H}$ for all ${\mathbf {y}}$.

To illustrate, suppose that there is a single known parameter $\mu$ and that phenotypes are distributed independently as $N\left( \mu ,\sigma ^{2}\right)$. Suppose that selection is such that only phenotypes under a threshold *t* are observed and that sample size is *N*; here, ${\varphi}=t$. The joint density of the observations, after selection, is:

4 $$p_{s}({\mathbf {y}}{{|}}t,\mu ,\sigma ^{2})=\frac{p({\mathbf {y}}{{|}}\mu ,\sigma ^{2})}{\int _{-\infty }^{t}p({\mathbf {y}}{{|}}\mu ,\sigma ^{2})d{\mathbf {y}}}=\frac{p({\mathbf {y}}{{|}}\mu ,\sigma ^{2})}{ { \prod \limits _{i=1}^{N}} \Pr (z_{i}<t)}=\frac{p({\mathbf {y}}{{|}}t,\mu ,\sigma ^{2})}{\overline{H}\left( t,\mu ,\sigma ^{2}\right) },$$

where $z_{i}=\dfrac{t_{i}-\mu }{\sigma }$ and $\overline{H}$ is the mean fitness, given $\mu$ and $\sigma ^{2}$. If a prior distribution *F* were assigned to $\mu ,$ Bayesian mean fitness would be $E_{F}\left[ \overline{H}\left( t,\mu ,\sigma ^{2}\right) \right] =\overline{H}\left( t,\sigma ^{2}\right) ,$ where the outer expectation indicates that the values of $\mu$ are averaged out using distribution *F* as mixing process. See [23] for an example of a discrete fitness function applied to the “cow setting” of [30], where fitness is equivalent to the probability of observing a certain pattern.

Therefore, the joint posterior density under selection is:

5 $$\begin{aligned} p_{s}({\theta},{{\varphi {|}}} {\mathbf{y}},Hyp,M)&\propto p_{s}({\mathbf {y}} ,{\theta},{\varphi}{{|}}Hyp,M) \\&\propto H({\mathbf {y}}{|}{\varphi},{\theta})p({\mathbf {y}}| {\theta},M)p({\theta},{\varphi}{{|}}Hyp,M) \\&\propto H({\mathbf {y}}{|}{\varphi},{\theta})p ({\mathbf {y}}|{\theta},M)p\left( {{\varphi}{|}{\theta},}M\right) p({\theta} {|}Hyp,M), \end{aligned}$$

where $p\left( {{\varphi}{|}{\theta},}M\right)$ is the conditional (given ${\theta}$) prior density of ${\varphi}$. If ${\varphi}$ and ${\theta}$ are independent a priori and if $H(\mathbf {y}|{\varphi }{,}{\theta})=H({\mathbf {y}}{|} {\varphi }),$ i.e., fitness does not depend on ${\theta},$ one has

6 $$p_{s}({\theta},{{\varphi {|}}} {\mathbf{y}},Hyp,M)\propto \left[ H({\mathbf {y}}{|} {\varphi})p\left( {\varphi}\right) \right] \left[ p({\mathbf {y}} |{\theta})p({\theta} {|}Hyp,M)\right] ,$$

implying that ${\theta}$ and ${\varphi}$ are also independent a posteriori. Therefore,

7 $$p_{s}({\theta} {|} {\mathbf{y}},Hyp,M)=p({{\theta}{|}{\mathbf {y}},} Hyp,M),$$

the posterior density of ${\theta}$ without selection. Thus, if selection is based on a fitness function (linear or non-linear) $H({\mathbf{y}}{|}{\varphi})$ of the observed data that does not depend on parameters ${\theta}$, and if ${\theta}$ and ${\varphi}$ are a priori independent, the selection process is ignorable from a Bayesian perspective. The posterior distributions before and after selection are exactly the same, in agreement with [22, 31, 32].

The preceding argument is at the root of the often-made claim that BLUP remains “unbiased if the history of the selection process is represented in the data used”, that is, if all records on which selection decisions were based are included in the analysis. The claim is not correct, at least from the frequentist perspective. Suppose ${\mathbf {y}}={\mathbf {g}}+{\mathbf {e}}$ is an *n*-dimensional vector in $R ^{n}$, where ${\mathbf {g}}{\sim}N\left( {\mathbf {0}},{\mathbf {G}}\right)$ is a vector of genetic effects and ${\mathbf {e}}{\sim}N\left( {\mathbf {0}} ,{\mathbf {R}}\right)$ is a vector of environmental deviates, with ${\mathbf {g}}$ independent of $\mathbf {e}.$ Selection is such that ${\mathbf {y}}{{{\in }}}S$ and *S* is the sampling space constrained by selection. If ${\mathbf {G}}$ and ${\mathbf {R}}$ are known, the posterior distribution of the genetic effects is $\mathbf {g}{|}\mathbf {G},\mathbf {R},\mathbf {y}{\sim}N\left(\widehat{{\mathbf {g}}},\left[ {\mathbf {R}} ^{-1}+{\mathbf {G}}^{-1}\right] ^{-1}\right) ,$ where the posterior expectation is $\widehat{{\mathbf {g}}}={\mathbf {G}}\left[ {\mathbf {R}}+{\mathbf {G}}\right] ^{-1}{\mathbf {y}}$ [31]. Following Eq. (7), the posterior distribution is unaffected by selection for any ${\mathbf {y}}$. Now, $\widehat{{\mathbf {g}}}$ is also a BLUP in a frequentist context and, in the absence of selection (${\mathbf{y}}{{{\in }}} R ^{n}$), $E\left( \widehat{{\mathbf {g}}}\right) ={\mathbf {G}}\left[ {\mathbf {R}}^{-1}+{\mathbf {G}}^{-1}\right] ^{-1}E\left( {\mathbf {y}}\right) =0$; hence, $E\left( \widehat{{\mathbf {g}}}\right) =E\left( {\mathbf {g}}\right)$ and the predictor is unbiased in the frequentist Hendersonian sense. Furthermore, $Var\left( \widehat{{\mathbf {g}}}\right) ={\mathbf {G}}\left[ {\mathbf {R}}+{\mathbf {G}}\right] ^{-1}{\mathbf {G}}$**.** However, if ${\mathbf {y}}{{{\in }}}S$,

8 $$E_{s}\left( \widehat{{\mathbf {g}}}\right) ={\mathbf {G}}\left[ {\mathbf {R}} ^{-1}+{\mathbf {G}}^{-1}\right] ^{-1}E_{s}\left( {\mathbf {y}}\right)$$

and

9 $$Var_{s}\left( \widehat{{\mathbf {g}}}\right) ={\mathbf {G}}\left[ {\mathbf {R}} ^{-1}+{\mathbf {G}}^{-1}\right] ^{-1}Var_{s}\left( {\mathbf {y}}\right) {\mathbf {G}}\left[ {\mathbf {R}}^{-1}+{\mathbf {G}}^{-1}\right] ^{-1},$$

since selection modifies the distribution of ${\mathbf {y}}$. Clearly, the distribution $\widehat{{\mathbf {g}}}\sim N\left( {\mathbf {0}},{\mathbf {G}}\left[ {\mathbf {R}}+{\mathbf {G}}\right] ^{-1}{\mathbf {G}}\right)$ is modified by the selection process. While the Bayesian treatment allows ignoring selection, a frequentist analysis requires finding the sampling distribution after selection. BLUP would be unbiased by selection only if it could be shown that $E_{s}\left( \widehat{{\mathbf {g}}}\right) =E_{s}\left( {\mathbf {g}}\right)$.

In a Bayesian treatment, the predictive distribution under selection of yet to be observed phenotypes ${\mathbf {y}}_{f}$ is

10 $$p_{s}\left( {\mathbf {y}}_{f}|{\mathbf {y}},Hyp,M\right) =\int p_{s}\left( {\mathbf {y}}_{f}|{\mathbf {y}},{{\theta},}M\right) p_{s}({\theta} {|} {\mathbf{y}} ,Hyp,M)d{\theta}.$$

If the process of generating future observations is unaltered by selection, $p_{s}\left( {\mathbf {y}}_{f}|{\mathbf {y}},{\theta},Hyp,M\right) =p\left( {\mathbf {y}}_{f}|{\mathbf {y}},{\theta},Hyp,M\right)$. Since the posterior distribution is unaffected by selection based on observed data, (10) can be re-written as:

11 $$p_{s}\left( {\mathbf {y}}_{f}|\mathbf {y},Hyp,M\right) =\int p\left({\mathbf {y}}_{f}|{\mathbf {y}},{\theta},M\right) p({\theta {|}} {\mathbf {y}}, Hyp,M)d{\theta}=p\left({\mathbf {y}}_{f}|\mathbf {y},Hyp,M\right).$$

Therefore, the predictive distribution is also unaltered by selection based on observed data. Prediction of future phenotypes can be carried out as if selection had not occurred. Hence, inferences from posterior or predictive probabilities are unaffected by this type of selection.

The same holds true for the posterior distribution of the model when treated as uncertain. Let there be *K* mutually exclusive and exhaustive competing models $M_{1},M_{2},\ldots,M_{K}$ with prior probabilities $P_{1},P_{2} ,\ldots,P_{K},$ and parameters ${\theta}_{1},{\theta}_{2},\ldots,{\theta}_{K},$ respectively. Under selection, the posterior probability assigned to model *k* is:

12 $$P_{s}\left( M_{k}|{\mathbf {y}},Hyp\right) =\frac{p_{s}\left( {\mathbf {y}} |M_{k},Hyp\right) P_{k}}{ { \sum \limits _{k=1}^{K}} p_{s}\left( {\mathbf {y}}|M_{k},Hyp\right) P_{k}};k=1,2,\ldots,K.$$

Above, $p_{s}\left( {\mathbf {y}}|M_{k},Hyp\right)$ is the marginal distribution of the data under model *k* with selection. Furthermore,

13 $$p_{s}\left( {\mathbf {y}}|M_{k},Hyp\right) =\int p_{s}\left( {\mathbf {y}} |{\theta}_{k}{,}M\right) p_{s}({\theta}_{k} {{|}}Hyp,M_{k})d{\theta}_{k}.$$

Using the reasoning employed for the predictive distribution, if $p_{s}\left( {\mathbf {y}}|{\theta}_{k}{,}M\right) =p\left( {\mathbf {y}} |{\theta}_{k}{,}M\right)$, then

14 $$p_{s}\left( {\mathbf {y}}|M_{k},Hyp\right) =\int p\left( {\mathbf {y}} |{\theta}_{k}{,}M\right) p({\theta}_{k}{|} Hyp,M)d{\theta}_{k}=p\left( {\mathbf {y}}|M_{k},Hyp\right) .$$

Using Eq. (14) in Eq. (12)

15 $$P_{s}\left( M_{k}|{\mathbf {y}},Hyp\right) =\frac{p\left( {\mathbf {y}} |M_{k},Hyp\right) P_{k}}{ { \sum \limits _{k=1}^{K}} p\left( {\mathbf {y}}|M_{k},Hyp\right) P_{k}}=P\left( M_{k}|{\mathbf {y}} ,Hyp\right) ;k=1,2,\ldots,K.$$

Therefore, the posterior distribution of the “model random variable” is also unaffected by selection and model choice can be carried out ignoring the selection process.

## Fitness based on observed and missing data

Inference under selection in quantitative genetics was formalized by Im et al. [22] by adapting missing data theory [21, 33] to maximum likelihood estimation of genetic parameters. In a study of the trajectory of genetic variance over time in a population undergoing selection, Sorensen et al [23] used such theory from a Bayesian perspective. To motivate this, let traits A and B be measured in *n* individuals, respectively, but 25% lack phenotypes for trait B. If the records in this portion are missing at random (e.g., it is expensive to record B and a random choice is made), there is no selection. What about if there is a non-random basis for the pattern of observed data? A connection between missingness and fitness is made below from a Bayesian perspective. The dependence on model *M* will be suppressed in the notation hereinafter.

A classical example dating back to 1959 [30], where selection is based on observed data only, serves to introduce the notion of missing data. Suppose two dairy cows have a first lactation record; only one of the cows will be allowed to produce a second lactation. Without selection, each of the cows would have milk records for each of the two lactations and the “complete” data would be ${\mathbf {y}}=\left( y_{11},y_{21},y_{12},y_{22}\right) ^{\prime },$ where *i* denotes cow and *j* record number. If $y_{11}>y_{21},$ the observed data would be ${\mathbf {y}}_{obs}=\left( y_{11},y_{21} ,y_{12}\right) ^{\prime };$ if $y_{11}\le y_{21}$ then ${\mathbf {y}} _{obs}=\left( y_{11},y_{21},y_{22}\right) ^{\prime }$. A vector ${\mathbf {r}}$ describing the pattern of “missingness” is part of the information on the problem. The two patterns are ${\mathbf {r}}=\left( 1,1,1,0\right) ^{\prime }$ and ${\mathbf {r}}=\left( 1,1,0,1\right) ^{\prime },$ respectively, where “$1$” is observed and “$0$” is missing. The complete data vector of phenotypes is ${\mathbf {y}}=\left( {\mathbf {y}}_{obs},{\mathbf {y}}_{miss}\right) ^{\prime }$ where ${\mathbf {y}}_{miss}$ includes the records that would have been observed if selection had not taken place.

Under selection, the joint density of all data (complete data and missingness pattern) and of the parameters is:

16 $$\begin{aligned}&p_{s}\left( {\mathbf {y}, \mathbf {r}},{\theta},{\varphi}{{|}}Hyp\right) \\&\quad =\Pr \left( {\mathbf {r}}{|}{\mathbf{y}}_{obs}{,}{\mathbf{y}} _{miss},{\theta},{\varphi}\right) p\left( {\mathbf{y}} _{obs}{,{\mathbf{y}}}_{miss}{|}{\theta}\right) p\left( {\theta},{\varphi}{{|}}Hyp\right) , \end{aligned}$$

where ${\varphi}$ are parameters of the missing data (selection) process and $\Pr \left( {\mathbf {r}}{|}{\mathbf{y}},{\theta},{\varphi}\right)$ is the conditional probability of observing the pattern. $\Pr \left( {\mathbf {r}}{{|}}{\mathbf{y}}_{obs}{,}{\mathbf{y}}_{miss}, {\theta},{{\varphi}}\right)$ is equivalent to the fitness function $H({\mathbf {y}}{{|}}$ ${{\varphi}}{,}{\theta})$ employed in Eq. (3) above and gives probabilities of “survival” (“death”), conditionally on phenotypes, observed and unobserved, and model parameters. Integrating with respect to ${\mathbf{y}}_{miss}$

17 $$\begin{aligned}&p_{s}\left( {\mathbf{y}}_{obs},{\mathbf {r}},{\theta}, {{{\varphi}}}{{|}}Hyp\right) \\&\quad =\int \Pr \left( {\mathbf {r}}{|}{\mathbf{y}}_{obs}{,{\mathbf{y}} }_{miss}{,{\theta},{{\varphi}}}\right) p\left( {\mathbf{y}} _{obs}{,{\mathbf{y}}}_{miss}{|}{\theta}\right) p\left( {\theta},{{\varphi}}{{|}}Hyp\right) d{\mathbf{y}} _{miss} \\&\quad =\int \Pr \left( {\mathbf {r}}{|}{\mathbf{y}}_{obs}{,{\mathbf{y}}}_{miss}{,{\theta}, {{\varphi}}}\right) p\left( {{\mathbf{y}} }_{miss}|{\mathbf{y}}_{obs},{\theta}\right) p\left( {\mathbf{y}}_{obs}{{|}{\theta}}\right) p\left( {{\theta} ,{{\varphi}}{|}}Hyp\right) d{\mathbf{y}}_{miss}. \end{aligned}$$

A rearrangment of the preceding equation leads to:

18 $$\begin{aligned}&p_{s}\left( {\mathbf{y}}_{obs},{\mathbf {r}},{\theta},{{\varphi}}{{|}}Hyp\right) \\&\quad \propto p\left( {\mathbf{y}}_{obs}{|}{\theta}\right) p\left( {{\theta},{{\varphi}}{|}}Hyp\right) \left[ \int \Pr \left( {\mathbf {r}}{|}{\mathbf{y}}_{obs}{,{\mathbf{y}}}_{miss}{,{\theta},{{\varphi}}} \right) p\left( {\mathbf{y}}_{miss} |{\mathbf{y}}_{obs},{\theta}\right) d{{{\mathbf {y}} }}_{miss}\right] . \end{aligned}$$

The term in brackets is the expected fitness after averaging over the conditional distribution of the missing data, given ${\mathbf{y}} _{obs}$, and ${\theta}{,}$ which we will denote as $H\left( {\mathbf{y}}_{obs},{{\varphi ,\theta }}\right) .$ Since $p\left( {\theta},{{\varphi}}{{|}}Hyp\right) =p\left( {\theta} {|}Hyp\right) p\left( {{{\varphi}}{|}{\theta}}\right)$ it follows that the posterior density under selection is:

19 $$\begin{aligned} p_{s}\left( {{\theta},{\varphi}{|}{\mathbf{y}}}_{obs},\mathbf {r}, Hyp\right) & \propto p\left( {\mathbf{y}}_{obs}{|{\theta}}\right) p\left({\theta {|}}Hyp\right) H\left({\mathbf {y}}_{obs},{{\varphi, \theta }}\right) p\left({{\varphi}{|}{\theta}}\right) \\ & \propto p\left( {{\theta}{|}{\mathbf{y}}}_{obs},Hyp\right) H\left({\mathbf {y}}_{obs},{\varphi,\theta}\right) p\left({\varphi}{|}{\theta}\right) . \end{aligned}$$

The preceding equation indicates that, in general, the posterior density under selection differs from the posterior density without selection. Correct Bayesian inference needs to take into account the selection process by formulating a selection model (i.e., defining a fitness function), as well as prior knowledge of $${{\varphi}}$$$${,}$$ deterministic or probabilistic. A selection model or $$H\left( {\mathbf{y}} _{obs},{{\varphi ,\theta }}\right)$$, must be specified, and a prior distribution of $${{\varphi}}$$ elicited or the value of this parameter specified *ex* *ante*.

A special case is when the fitness function does not involve missing data and parameters ${\theta}$, so $\Pr \left( {\mathbf {r}}{|}{\mathbf {y}} _{obs},{\mathbf{y}}_{miss},{\theta},{{\varphi}}\right) =\Pr \left( {\mathbf {r}}{|}{\mathbf{y}}_{obs},{{{\varphi}}}\right) .$ Here, the integral in (18) produces:

20 $$H\left( {\mathbf{y}}_{obs},{{\varphi}},{\theta}\right) =\int \Pr \left( {\mathbf {r}}{|}{\mathbf{y}}_{obs}{,{{\varphi}}}\right) p\left( {\mathbf{y}}_{miss}|{\mathbf{y}}_{obs},{\theta}\right) d {\mathbf{y}}_{miss}=\Pr \left( {\mathbf {r}}{|}{\mathbf{y}}_{obs}{,{{\varphi}}}\right) ,$$

since $p\left( {\mathbf{y}}_{miss}|{\mathbf{y}} _{obs},{\theta}\right)$ integrates to 1. Then, Eq. (19) becomes:

21 $$p_{s}\left( {\theta},{{\varphi {|}}} {\mathbf{y}}_{obs},{\mathbf {r}},Hyp\right) \propto p\left( {{\theta}{|}{\mathbf {y}}}_{obs},Hyp\right) \Pr \left( {\mathbf {r}}{|}{\mathbf{y}}_{obs}{,{{\varphi}}}\right) p\left( {{{\varphi}} {|}{\theta}}\right) .$$

Furthermore, if ${{\varphi}}$ and ${\theta}$ are independent a priori $p\left( {{{\varphi}}{|}{\theta}}\right) =p\left( {{{\varphi}}}\right) ,$ then:

22 $$\begin{aligned} p_{s}\left( {\theta},{{\varphi {|}} }{\mathbf{y}}_{obs},{\mathbf {r}},Hyp\right)&\propto p\left( {{\theta}{|}{\mathbf {y}}}_{obs},Hyp\right) \Pr \left( {\mathbf {r}}{|}{\mathbf{y}}_{obs},{{\varphi}}\right) p\left( {{\varphi}}\right) \\&\propto p\left( {{\theta}{|}{\mathbf {y}}}_{obs},Hyp\right) p\left( {{{\varphi}}{|}{\mathbf {y}}}_{obs},{\mathbf {r}}\right) , \end{aligned}$$

where

23 $$p\left( {{{\varphi}}{|}{\mathbf {y}}}_{obs},{\mathbf {r}}\right) =\frac{\Pr \left( {\mathbf {r}}{|}{\mathbf{y}}_{obs}{,{{\varphi}}}\right) p\left( {{\varphi}}\right) }{\int \Pr \left( {\mathbf {r}}{|}{\mathbf{y}}_{obs} ,{{\varphi}}\right) p\left( {{\varphi}}\right) d{{\varphi}}}$$

is the posterior density of fitness function parameter ${{\varphi}}.$ Expression (22) implies that ${\theta}$ and ${{\varphi}}$ are also independent a posteriori. Hence, $p_{s}\left( {\theta} {|} {\mathbf{y}}_{obs},\mathbf {r},Hyp\right) =p\left( {\theta}{|}{\mathbf{y}}_{obs},Hyp\right)$, so Bayesian inference about ${\theta}$ can be carried out as if selection had not taken place, irrespective of the pattern of missingness created by selection on ${\mathbf{y}}_{obs}$. In other words: if the conditional probability of observing a phenotype (“fitness”) depends on observed data but not on missing data and on ${\theta}{,}$ and if parameters ${\theta}$ and ${{\varphi}}$ are independent *a priori * (note that Im et al. [22] used the term “distinct” in their likelihood-based treatment), selection can be ignored. The two conditions, however, represent strong assumptions. Equation (19) indicates that even if ${\theta}$ and ${{\varphi}}$ are assigned independent prior distributions, selection cannot be ignored in inference any time that the fitness function involves ${\theta}{,}$ i.e., if it has the form $H\left( {\mathbf{y}}_{obs},{{\varphi ,\theta }}\right)$ as opposed to $H\left( {\mathbf{y}}_{obs},{{\varphi}}\right)$.

In spite of the strong assumptions required, most animal breeding programs employ statistical methods that ignore selection coupled with data sets that do not reflect the entire history of the selection process. Clearly, there are enormous difficulties in representing the type of selection undergoing in populations of animals. Generations overlap, animals have unequal amounts of information and of relatedness to other animals, and it is not always transparent why certain individuals are kept as parents even when recording is complete and meticulous. A cautionary view may be that inference of breeding values is always distorted (often loosely referred to as “bias”) to some extent due to various factors that cannot be accounted for statistically. Viewing estimates as being free from selection bias is perhaps naïve.

## Selection and partially observed data

Consider (19) placing focus on the fitness function $H\left( {\mathbf{y}}_{obs},{{\varphi ,\theta }}\right)$ defined in (20). The distribution $\left[ {{\mathbf{y}} }_{miss}|{\mathbf{y}}_{obs},{\theta}\right]$ follows from the statistical model assumed. For example, if the joint distribution of ${\mathbf{y}}_{miss}$ and ${\mathbf{y}}_{obs}$, given ${\theta}$, is normal, the conditional distribution is normal as well, with mean vector and covariance matrix readily derived from theory. Next, one would need to assume a selection model representing the distribution of the missing data pattern $\left( {\mathbf {r}}\right) .$ Four examples are presented below to illustrate concepts, motivated by those described in [22] and adapted to a Bayesian perspective.

### Example 1: selection based on records not available for analysis

Country B buys frozen semen of *m* out of *n* bulls $\left( m<n\right)$ in country A. The *m* bulls chosen exceed a minimum threshold of “performance” *t* based on information provided by country A. Country B develops a breeding program using such *m* bulls and collects records. Only records from country B are available for analysis. The performances in the two countries are regarded as distinct traits [34], a concept that has been employed in global dairy cattle breeding.

Assuming conditional (given ${\theta}$) independence between records, the data generating model for the *m* records observed in country B is:

24 $$p\left( {\mathbf {y}}_{obs}|{\theta}\right) = { \prod \limits _{i=1}^{m}} p\left( y_{iB}|{\theta}\right) ,$$

where $y_{iB}$ is the performance of bull *i* ($i=1,2,\ldots,m$). Following [22], the conditional probability of selection involves two binary indicator variables, $r_{iA}$ and $r_{iB},$ where the values 0 and 1 mean “missing” and “observed”, respectively. For $i=1,2,\ldots,n,$ where *n* (number of bulls in country A),

25 $$\Pr \left( r_{iA}=0|{\mathbf {y}}_{obs},{\mathbf {y}}_{miss}\right) =1\text { and }\Pr \left( r_{iB}=1|{\mathbf {y}}_{obs},{\mathbf {y}}_{miss}\right) =1_{\left( t,\infty \right) }\left( y_{iA}\right) .$$

The value $r_{iA}=0$ for all *i* means that all records from the exporting country (A) are not available (“missing”); $r_{iB}=1$ means that records on bull *i* are observed in country B only if selection threshold *t* is exceeded by such bull in country A.

The density of all data (observed and missing) and of ${\mathbf {r}}$, is:

26 $$p\left( {\mathbf {y}}_{obs},{\mathbf {y}}_{miss},{\mathbf {r}}|{\theta}\right) = {\prod \limits _{i=1}^{m}} p\left( y_{iA},y_{iB}|{\theta}\right) 1_{\left( t,\infty \right) }\left( y_{iA}\right) {\prod \limits _{i=m+1}^{n}} p\left( y_{iA},y_{iB}|{\theta}\right) 1_{\left( -\infty ,t\right) }\left( y_{iA}\right) .$$

Missing data includes all *n* records from country A, and the $n-m$ records that would have been observed in country B but were not because the corresponding bulls did not perform over threshold *t* in A. Integrating Eq. (26) with respect to the missing data yields:

27 $$\begin{aligned} & p\left( {\mathbf {y}}_{obs},{\mathbf {r}}|{\theta}\right) \\&=\left\{ { \prod \limits _{i=1}^{m}} { \int } p\left( y_{iA},y_{iB}|{\theta}\right) 1_{\left( t,\infty \right) }\left( y_{iA}\right) dy_{iA}\right\} \left\{ { \prod \limits _{i=m+1}^{n}} { \iint } p\left( y_{iA},y_{iB}|{\theta}\right) 1_{\left( -\infty ,t\right) }\left( y_{iA}\right) dy_{iA}dy_{iB}\right\} \\& = { \prod \limits _{i=1}^{m}} p\left( y_{iB}|{\theta}\right) \left[ { \prod \limits _{i=1}^{m}} \Pr \left( y_{iA}>t|y_{iB},{\theta}\right) { \prod \limits _{i=m+1}^{n}} \Pr \left( y_{iA}<t|{\theta}\right) \right] . \end{aligned}$$

Using observed data does not make use of the information on the selection process provided by the two terms between brackets.

Here, ${{\varphi}}=t$ (known) is the only parameter that governs the missing data process. The posterior density after accounting for selection is:

28 $$\begin{aligned}&p\left( {\theta} {|} {\mathbf{y}}_{obs},\mathbf {r}, Hyp\right) \\&\propto { \prod \limits _{i=1}^{m}} p\left( y_{iB}|{\theta}\right) p\left( {\theta} {|}Hyp\right) \left[ { \prod \limits _{i=1}^{m}} \Pr \left( y_{iA}>t|y_{iB},{\theta}\right) { \prod \limits _{i=m+1}^{n}} \Pr \left( y_{iA}<t|{\theta}\right) \right] \\&\propto p\left( {\theta} {|} {\mathbf{y}}_{obs},Hyp\right) H\left( {\mathbf {y}}_{obs},t,{\theta}\right) , \end{aligned}$$

where $H\left( {\mathbf {y}}_{obs},t,{\theta}\right)$ is the term in brackets.

This simple selection scheme produces an analytically intractable problem. In the absence of selection, let performances in countries A and B have the bivariate distribution:

29 $$\left[ \begin{array}{c} y_{iA}\\ y_{iB} \end{array} \right] |\mu _{A},\mu _{B},{{\Sigma}{\sim}}N\left( \left[ \begin{array}{c} \mu _{A}\\ \mu _{B} \end{array} \right] ,\left[ \begin{array}{cc} \sigma _{A}^{2} & \rho \sigma _{A}\sigma _{B}\\ \rho \sigma _{A}\sigma _{B} & \sigma _{B}^{2} \end{array} \right] \right) ;i=1,2, \ldots n.$$

Assume such vectors are mutually independent, with $\mu _{A}$ and $\mu _{B}$ being country means, $\rho$ the correlation coefficient between performances, and $\sigma _{A}^{2}$ and $\sigma _{B}^{2}$ the variances in countries A and B, respectively. Suppose all parameters are known, save for $\mu _{B}$, the mean performance in the importing country. The conditional distribution $\left[ y_{iA}|y_{iB},{\theta}\right]$ is:

30 $$y_{iA}|y_{iB},{{\theta}{{\sim }}}N\left( \mu _{A.B}=\mu _{A}+b\left( y_{iB}-\mu _{B}\right) ,v_{A.B}=\sigma _{A}^{2}\left( 1-\rho ^{2}\right) \right) ;$$

above, $b_=\dfrac{\rho \sigma _{A}\sigma _{B}}{\sigma _{B}^{2}}$ is the regression of performance in A on that in B. Using (28) and assigning a flat prior to $\mu _{B}$, the posterior density is:

31 $$\begin{aligned} p\left( \mu _{B}{|}{\mathbf{y}}_{obs},\mathbf {r},Hyp\right)&\propto \exp \left[ -\frac{ { \sum \limits _{i=1}^{m}} \left( y_{iB}-\mu _{B}\right) ^{2}}{2\sigma _{B}^{2}}\right] { \prod \limits _{i=1}^{m}} \left[ 1-\Phi _{i,A.B}\left( t\right) \right] { \prod \limits _{i=m+1}^{n}} \Phi _{i,A}\left( t\right) \\&\propto \exp \left[ -\dfrac{ { \sum \limits _{i=1}^{m}} \left( y_{iB}-\mu _{B}\right) ^{2}}{2\sigma _{B}^{2}}\right] { \prod \limits _{i=1}^{m}} \left[ 1-\Phi _{i,A.B}\left( t\right) \right] , \end{aligned}$$

where $\Phi _{i,A.B}$ is the distribution function of Eq. (30), which depends on $\mu _{B}.$ The expression involving $\Phi _{i,A}$, the distribution function of performance in country A, is absorbed by the integration constant. If selection is ignored, the posterior distribution would be proportional to the Gaussian kernel above with mean (mode) ${\widehat{\mu }}_{B}=\dfrac{ { \sum \limits _{i=1}^{m}} y_{iB}}{m}.$ Under selection, however, the posterior mean cannot be written in closed form and locating the mode requires an iterative procedure. If $\sigma _{A}^{2}=\sigma _{B}^{2}=1$ and $\mu _{A}=0,$ for instance, the posterior density takes the form:

32 $$p\left( \mu _{B}{|}{\mathbf{y}}_{obs},\mathbf {r},Hyp\right) =\frac{\exp \left\{ -\frac{1}{2}\left[ m\left( \mu _{B}-{\widehat{\mu }}_{B}\right) ^{2}-2f\left( \mu _{B}\right) \right] \right\} }{ { \int } \exp \left\{ -\frac{1}{2}\left[ m\left( \mu _{B}-{\widehat{\mu }}_{B}\right) ^{2}-2f\left( \mu _{B}\right) \right] \right\} d\mu _{B}}$$

where

33 $$f\left( \mu _{B}\right) = { \sum \limits _{i=1}^{m}} \log \left[ 1-\Phi _{i}\left( \dfrac{t-\rho \left( y_{iB}-\mu _{B}\right) }{\sqrt{1-\rho ^{2}}}\right) \right] .$$

**Fig. 1:**
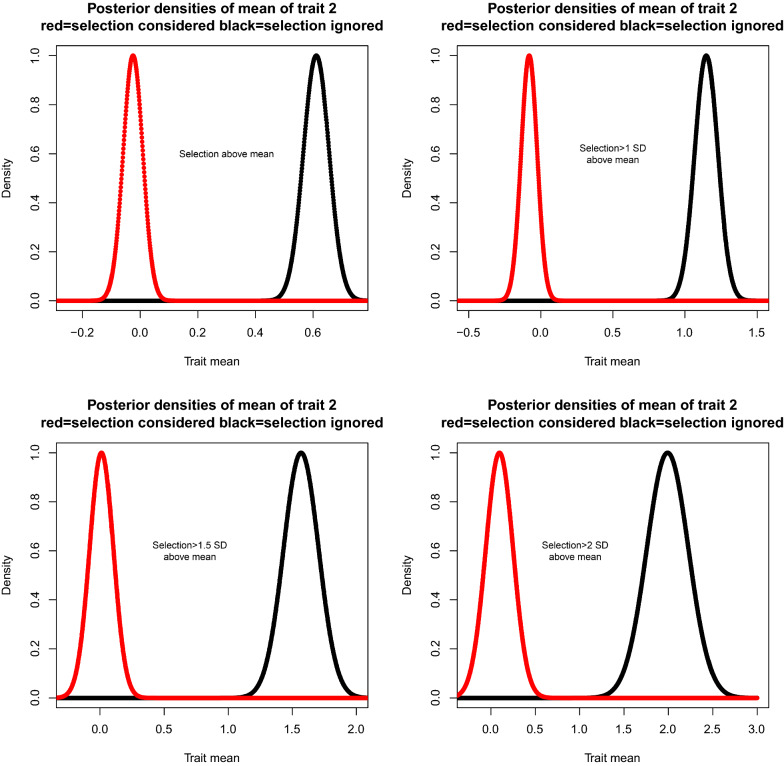
Posterior distribution: selection for correlated traits at various intensities

To illustrate how accounting for selection may lead to correct Bayesian inference, we simulated $n=1000$ pairs from a bivariate standard normal distribution with $\rho =0.8$. Selection operated on trait A by picking individuals with phenotypes that were above the mean or 1, 1.5 and 2 standard deviations over the mean. Such selection produced samples of sizes $m=494,155,53$ and 18 on which performance for trait *B* was available. The only parameter treated as unknown was $\mu _{B}$ with a flat prior attached to it. If selection were ignored, the posterior distribution would be $\mu _{B}|y_{B}\sim N\left( {\widehat{\mu }}_{B},m^{-1}\right) ;$ if selection is accounted for, the posterior density is as in (32). On the one hand, Fig. 1 shows that ignoring selection grossly overstated the true value of the parameter: 0. On the other hand, the true value was assigned appreciable density in the “correct” posterior distributions, irrespective of the selection intensity applied. Note that the fitness function (missing data process) employed corresponds exactly to how selection was simulated. An incorrect formulation of the selection process would have probably produced distorted inferences. The example illustrates that a proper Bayesian analysis may capture true parameter values in situations of non-ignorable selection when the latter is modeled properly.

### Example 2: pre-selected samples

The example is motivated by a situation in animal and plant breeding that has taken place during the genomic era and that it produces what is called “pre-selection bias”. It was studied by Patry and Ducrocq [35] in a dairy cattle setting but from a different perspective to the one employed in our paper. In New Zealand dairy cattle, Winkelman et al. [36] proposed a method of genetic evaluation that combined a Gaussian kernel that is constructed using genomic information with features of single-step BLUP methodology. The procedure did not use the notions of missing data or of fitness functions. With real records, they found that the proposed methodology delivered a smaller predictive bias and a higher predictive correlation than a previously used procedure that “blended” pedigree and genomic information. The correlation improved 1–2% for production traits, but negligibly for traits such as fertility or longevity. In a simulation study [37], produced 15 generations of selection. At that point, parents were preselected with various degrees of intensity using either parental averages, a genome-based choice, or at random. Subsequently, they estimated genomic breeding values of preselected animals with a single-step BLUP procedure that excluded all the information from pre-culled individuals. They were not able to detect bias in the evaluations of such animals. Wang et al. [38] considered the impact of genomic selection on estimates of variance components obtained from using different similarity kernels, and used simulation and real data from broilers. When genotyping was at random, estimates obtained with a single-step model did not exhibit bias in the simulated data sets; otherwise, estimates had a marked bias. The impact of such bias on estimated breeding values was noticeable. It is unclear to what extent the results from these three studies generalize to more general forms of selection, as consideration of a general prescription was not addressed. Simulations provide “local” guidance only: results may change drastically if the assumptions adopted or the structure of the data are varied. These researchers, however, seemed aligned with the view that accounting for the history of the selection process as completely as possible can attenuate the impact of selection on inference and prediction, rendering selection quasi-ignorable.

When genomic selection began to be applied [10], decisions had to be made on individuals (e.g., bulls) to be genotyped for single nucleotide polymorphisms (SNPs). Due to the high cost of SNP chips, not all candidates for selection could be genotyped; a similar situation occurs now with next-generation DNA sequences or with expensive epigenomic or metabolomic measurements. Suppose that *m* out of *n* ($m<n$) dairy bulls that possess pedigree-based estimates of breeding value are chosen for genotyping, with “genomic breeding values” estimated as if the *m* bulls were randomly sampled. A “pre-selection bias” is expected to accrue since the *m* bulls chosen may not be representative of the current population. Can the distortion in inference be tempered analytically?

For illustration, a wheat yield data set examined in several studies and available in the BGLR package was used [39–41]. The dataset spans $n=599$ inbred lines of wheat, each genotyped for $p=1279$ binary markers that denote presence or absence of an allele at a locus. The target phenotype was wheat grain yield in each line planted in “environment 1”. The dataset also includes a pedigree-based additive relationship matrix, ${\mathbf {A}},$ of size 599 $\times$ 599 and several of the lines are completely inbred. A genomic relationship matrix [17] among lines was built as ${\mathbf {G}}=\mathbf {XX}^{\prime }/p$ where ${\mathbf {X}}$ was the $599\times 1279$ matrix of marker codes (0,1) with each column centered and standardized. Using the entire dataset, i.e., without any selection practiced, genetic (pedigree or genome based) and residual variance components were estimated via maximum likelihood. The random effects models used were ${\mathbf {y}}={\mathbf {a}}+{\mathbf {e}}$ and ${\mathbf {y}}={\mathbf {g}}+{\mathbf {e}} ^{*}$ in pedigree-based and genome-enabled analyses, respectively, where ${\mathbf {a}}$ and ${\mathbf {g}}$ are pedigree and genomic breeding values to be learned, with ${\mathbf {e}}$ and ${\mathbf {e}}^{*}$ being model residuals. We posed $\mathbf {a|}\sigma _{a}^{2}\sim N\left( {\mathbf {0}},{\mathbf {A}}\sigma _{a}^{2}\right) ,$ $\mathbf {e|}\sigma _{e}^{2}\sim N\left( {\mathbf {0}} ,{\mathbf {I}}\sigma _{e}^{2}\right)$ as mutually independent, and likewise for ${\mathbf {g}}{{|}}\sigma _{g}^{2}\sim N\left( {\mathbf {0}},{\mathbf {G}}\sigma _{g} ^{2}\right) ,$ ${\mathbf {e}}^{*}{{|}}\sigma _{e^{*}}^{2}\sim N\left( {\mathbf {0}},{\mathbf {I}}\sigma _{e^{*}}^{2}\right) ;$ the $\sigma ^{2\prime }s$ are variance components. Using the maximum likelihood estimates of variances as true values, best linear unbiased predictions of ${\mathbf {a}}$ and ${\mathbf {g}}$ were calculated, $\widehat{{\mathbf {a}}}$ and $\widehat{{\mathbf {g}} },$ respectively. Under a Bayesian framework the posterior distributions of the pedigree and genomic breeding values are ${\mathbf {a}}{|}{\mathbf{y}},\sigma _{a} ^{2},\sigma _{e}^{2}{\sim}N\left( \widehat{{\mathbf {a}}},{\mathbf {C}} _{a}\right)$ and ${\mathbf {g}}{|}{\mathbf {y}},\sigma _{g}^{2},\sigma _{e^{*}} ^{2}{\sim}N\left( \widehat{{\mathbf {g}}},\mathbf {C}_{g}\right)$, with variance components treated as known hyper-parameters. Here,

34 $$\widehat{{\mathbf {a}}}=\left( {\mathbf {I}}+\frac{\sigma _{e}^{2}}{\sigma _{a}^{2} }{\mathbf {A}}^{-1}\right) ^{-1}{\mathbf {y}}\text { and }\widehat{{\mathbf {g}} }=\left( {\mathbf {I}}+\frac{\sigma _{e^{*}}^{2}}{\sigma _{g}^{2}} {\mathbf {G}}^{-1}\right) ^{-1}\mathbf {y},$$

give the posterior expectations and

35 $${\mathbf {C}}_{a}=\left( {\mathbf {I}}+\frac{\sigma _{e}^{2}}{\sigma _{a}^{2} }{\mathbf {A}}^{-1}\right) ^{-1}\sigma _{e}^{2}\text { and }{\mathbf {C}}_{g}=\left( {\mathbf {I}}+\frac{\sigma _{e^{*}}^{2}}{\sigma _{g}^{2}}{\mathbf {G}}^{-1}\right) ^{-1}\sigma _{e^{*}}^{2},$$

are the posterior covariance matrices. In a frequentist setting, the posterior means correspond to $BLUP\left( {\mathbf {g}}\right)$ and $BLUP\left( {\mathbf {a}}\right) ,$ whereas ${\mathbf {C}}_{a}$ and ${\mathbf {C}}_{g}$ are interpreted as prediction error covariance matrices.

**Fig. 2:**
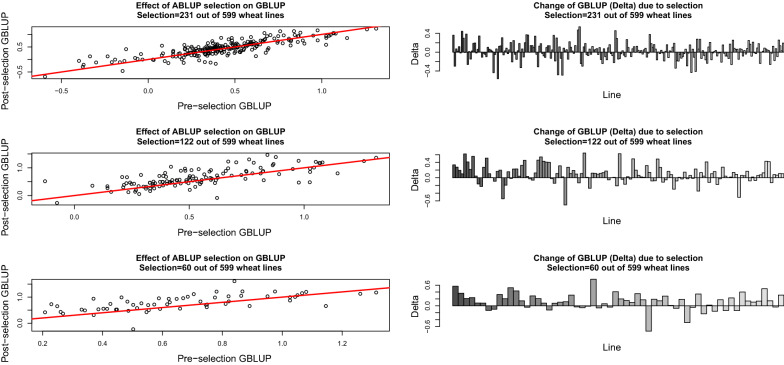
Effect of selection on posterior means of pedigree (ABLUP) and genome-based (GBLUP) breeding values

Using the 599 values in $\widehat{{\mathbf {a}}},$ we selected lines with pedigree breeding value estimates larger than the threshold $t=$ 0.20, 0.40 or 0.60, resulting in 231, 122 and 60 “top” lines, respectively. The posterior distributions of ${\mathbf {g}}$ were calculated before and after selection (ignoring the missing data process but using the same variance components) and compared. As depicted in Fig. 2, the analysis based on selected lines tended to overstate estimates of genomic breeding values relative to those obtained without selection. Ignoring selection introduces a selection “bias” that is impossible to evaluate because the true breeding values are unknown, except when data are simulated. Letting $M_{a}$ and $M_{g}$ denote pedigree and genome-based models, respectively, note that

36 $$\begin{aligned} E\left( \widehat{{\mathbf {a}}}|\mathbf {a},M_{a}\right)&=\left( {\mathbf {I}}+\frac{\sigma _{e}^{2}}{\sigma _{a}^{2}}{\mathbf {A}}^{-1}\right) ^{-1}E\left( {\mathbf {y}}|{\mathbf {a}}\right) =\left( {\mathbf {I}}+\frac{\sigma _{e}^{2}}{\sigma _{a}^{2}}{\mathbf {A}}^{-1}\right) ^{-1}\mathbf {a}, \end{aligned}$$

37 $$\begin{aligned} E\left( \widehat{{\mathbf {g}}}|\mathbf {g},M_{g}\right)&=\left( {\mathbf {I}}+\frac{\sigma _{e^{*}}^{2}}{\sigma _{g}^{2}}{\mathbf {G}}^{-1}\right) ^{-1}E\left( {\mathbf {y}}|{\mathbf {g}}\right) =\left( {\mathbf {I}}+\frac{\sigma _{e^{*}}^{2}}{\sigma _{g}^{2}}{\mathbf {G}}^{-1}\right) ^{-1} \mathbf {g}. \end{aligned}$$

Hence, both $\widehat{{\mathbf {a}}}$ and $\widehat{{\mathbf {g}}}$ have an “epistemic” bias [24]. Such bias differs from the notion used by Henderson [42, 43], who defined “prediction unbiasedness” as $E\left( \widehat{{\mathbf {a}}}\right) =E\left( {\mathbf {a}}\right)$ under $M_{a}$ or $E\left( \widehat{{\mathbf {g}}}\right) =E\left( {\mathbf {g}}\right)$ under $M_{g}$, i.e., posterior means (BLUP) are unbiased for the mean of the prior distributions, but not for the estimands ${\mathbf {a}}$ and ${\mathbf {g}}$. Selection introduces an additional distortion relative to “true” breeding values, pedigree or genome-defined.

How does one account for the distortion in inference? Our representation of selection will follow the protocol employed in the example. Threshold *t* is the only parameter governing selection here. Let “*sel*” and “*nsel*” denote selected and unselected individuals, respectively. Following (28), the posterior density of the genomic breeding values after accounting for selection and assuming that $\left[ \widehat{{\mathbf {a}}}|\mathbf {y,g}\right] =\left[ \widehat{{\mathbf {a}}}|{\mathbf {g}}\right]$ (i.e., given $\mathbf {g},$ $\widehat{{\mathbf {a}}}$ is independent of ${\mathbf {y}}$), one has:

38 $$\begin{aligned}&p\left( \mathbf {g|y}_{obs},\mathbf {r},Hyp\right) \\&\quad \propto { \prod \limits _{i=1}^{m}} p\left( y_{i}|g_{sel,i}\right) p\left( \mathbf {g|}Hyp\right) \left[ { \prod \limits _{i=1}^{m}} \Pr \left( {\widehat{a}}_{sel,i}>t|{\mathbf {g}}_{sel}\right) { \prod \limits _{i=m+1}^{n}} \Pr \left( {\widehat{a}}_{nsel,i}<t|{\mathbf {g}}_{nsel}\right) \right] \\&\quad \propto \left[ { \prod \limits _{i=1}^{m}} p\left( y_{i}|g_{sel,i}\right) p\left( {\mathbf {g}}_{sel}{|} Hyp\right) \right] \times \left[ { \prod \limits _{i=1}^{m}} \Pr \left( {\widehat{a}}_{sel,i}>t|g_{sel,i}\right) \right. \\&\quad \left. { \prod \limits _{i=m+1}^{n}} \Pr \left( {\widehat{a}}_{nsel,i}<t|g_{nsel,i}\right) \right] p\left( {\mathbf {g}}_{nsel}\mathbf {|g}_{sel},Hyp\right) . \end{aligned}$$

The first term in brackets is the posterior density of the genomic breeding values calculated from selected data only but ignoring selection. The joint fitness function [second term in brackets in (38)] assumes that the choice of an individual for genotyping is based only on whether or not *t* is exceeded, independently of what happens with other individuals, but conditionally on the unknown genomic breeding value of the individual in question. Integrating (38) with respect to ${\mathbf {g}}_{nsel}$ produces

39 $$\begin{aligned}&p\left( {\mathbf {g}}_{sel}{|}{\mathbf{y}}_{obs},\mathbf {r},Hyp\right) \\&\propto p\left( {\mathbf {g}}_{sel}{|}{\mathbf{y}}_{obs},Hyp\right) { \prod \limits _{i=1}^{m}} \Pr \left( {\widehat{a}}_{sel,i}>t|g_{sel,i}\right) \\&\times { \int } { \prod \limits _{i=m+1}^{n}} \Pr \left( {\widehat{a}}_{nsel,i}<t|g_{nsel,i}\right) p\left( {\mathbf {g}} _{nsel}\mathbf {|g}_{sel},Hyp\right) d{\mathbf {g}}_{nsel}. \end{aligned}$$

Since unselected individuals are not genotyped, there is no information available for writing $p\left( {\mathbf {g}}_{nsel}\mathbf {|g}_{sel},Hyp\right) ,$ which is Gaussian with mean ${\mathbf {G}}_{nsel,sel}{\mathbf {G}}_{sel,sel} ^{-1}{\mathbf {g}}_{sel}$ and covariance matrix ${\mathbf {G}}_{nsel,nsel} -{\mathbf {G}}_{nsel,sel}{\mathbf {G}}_{sel,sel}^{-1}{\mathbf {G}}_{sel,nsel}$. Hence the integral in (39) does not convey information on ${\mathbf {g}}_{sel}$ and is treated as a constant. The preceding is an important matter and may adversely affect the ability of accounting for selection. Finally,

40 $$p\left( {\mathbf {g}}_{sel}{|}{\mathbf{y}}_{obs},\mathbf {r},Hyp\right) \propto \exp \left[ -\frac{1}{2\sigma _{e^{*}}^{2}}\left( {\mathbf {g}}_{sel} -\widetilde{{\mathbf {g}}}_{sel}\right) ^{\prime }\widetilde{{\mathbf {C}}} _{g,sel}^{-1}\left( {\mathbf {g}}_{sel}-\widetilde{{\mathbf {g}}}_{sel}\right) +f_{sel}\left( {\mathbf {g}}_{sel}\right) \right] ,$$

where,

41 $$\widetilde{{\mathbf {g}}}_{sel}=\widetilde{{\mathbf {C}}}_{g,sel}{\mathbf {y}}_{sel},$$

42 $$\widetilde{{\mathbf {C}}}_{g,sel}=\left[ {\mathbf {I}}_{sel,sel}+\frac{\sigma _{e^{*}}^{2}}{\sigma _{g}^{2}}{\mathbf {G}}_{sel,sel}^{-1}\right] ^{-1},$$

and

43 $$f_{sel}\left( {\mathbf {g}}_{sel}\right) = { \sum \limits _{i=1}^{m}} \log \left[ \Pr \left( {\widehat{a}}_{sel,i}>t|g_{sel,i}\right) \right] .$$

We note that it might be possible to approximate the distribution $\left[ {\mathbf {g}}_{nsel}\mathbf {|g}_{sel},Hyp\right]$ by making an imputation from pedigree information, as in single-step methods [44]; however, this is a technical matter beyond the scope of our paper.

Simplifying assumptions are required in order to proceed. A canonical case is one where individuals are independently and identically distributed. Without selection, let

44 $$\left[ \begin{array}{c} g_{i}\\ {\widehat{a}}_{i} \end{array} \right] \sim N\left( \left[ \begin{array}{c} 0\\ 0 \end{array} \right] ,\left[ \begin{array}{cc} \sigma _{g}^{2} & \rho _{g{\widehat{a}}}\sigma _{g}\sigma _{{\widehat{a}}}\\ \rho _{g{\widehat{a}}}\sigma _{g}\sigma _{{\widehat{a}}} & \sigma _{{\widehat{a}}}^{2} \end{array} \right] \right) ;i=1,2,\ldots,n,$$

where $\rho _{g{\widehat{a}}}$ is the expected correlation between unknown genomic breeding value and pedigree-based posterior mean (BLUP here) and the $\sigma ^{2}$ are variance parameters. In real applications $\rho _{g{\widehat{a}}}$ actually varies over candidates due to unequal amounts of information. In the simplest case, $\sigma _{{\widehat{a}}}=\sqrt{Var\left( h_{a}^{2}y_{i}\right) }=h_{a}^{2}\sqrt{\sigma _{a}^{2}+\sigma _{e}^{2}}$, where $\sigma _{a}^{2}$ and $\sigma _{e}^{2}$ pertain to the pedigree-based model and $h_{a}^{2}=\dfrac{\sigma _{a}^{2}}{\sigma _{a}^{2}+\sigma _{e}^{2}}$ is heritability. Then, $E\left( {\widehat{a}}_{i}|g_{i}\right) =\rho _{g{\widehat{a}}}\dfrac{\sigma _{{\widehat{a}}}}{\sigma _{g}}g_{i},$ and $Var\left( {\widehat{a}}_{i}|g_{i}\right) =h_{a}^{4}\left( \sigma _{a}^{2}+\sigma _{e} ^{2}\right) \left( 1-\rho _{g{\widehat{a}}}^{2}\right)$ for all *i*. Dispersion parameter estimates were $\sigma _{a}^{2}=0.2859,\sigma _{e} ^{2}=0.5761,h_{a}^{2}=0.3316$ and $\sigma _{g}^{2}=0.5315$. Since there is no information on $\rho _{g{\widehat{a}}}$, using the unselected data we crudely estimated $\rho _{{\widehat{g}}{\widehat{a}}}$ at 0.82 and took $\rho _{g{\widehat{a}}}=0.75$ for the example. In order to account somehow for the fact that individuals were not identically distributed, the following modifications of the previous formulae were made using BLUP theory: $\sigma _{{\widehat{a}}}\rightarrow \sqrt{\sigma _{{\widehat{a}}_{i}}^{2}}$; $\sigma _{g}\rightarrow \sqrt{\sigma _{g_{i}}^{2}}$ and $Var\left( {\widehat{a}}_{i}|g_{i}\right) =\sigma _{{\widehat{a}}_{i}}^{2}\left( 1-\dfrac{\sigma _{{\widehat{a}}_{i}}^{2}}{\sigma _{g_{i}}^{2}}\right)$ for $i=1,2,\ldots,n$. Here, for example, $\rho _{g_{i}{\widehat{a}}_{i}}$ is the correlation value specific to individual *i* and $\sigma _{g_{i}}$ is the square root of the appropriate diagonal element of ${\mathbf {G}}\sigma _{g}^{2}$. The posterior density of genomic breeding values after selection was therefore:

45 $$\begin{aligned} & p \left({\mathbf {g}}_{sel} | {\mathbf{y}}_{obs},{\mathbf{r}}, {Hyp}\right) \\ & \quad \propto \exp \left\{ -\frac{{1}}{{2\sigma_{e^*}^{2}}} \left({\mathbf{g}}_{sel} - {\widetilde{\mathbf{g}}}_{sel} \right)^{\prime} {\widetilde{\mathbf{C}}}_{g,sel}^{-1} \left( {\mathbf{g}}_{sel} -{\widetilde{\mathbf{g}}}_{sel}\right) + \sum\limits_{i=1}^{m} \log [ 1-\Phi ( z_{i}) ] \right\} , \end{aligned}$$

where

46 $$z_{i}=\frac{t-\rho _{g_{i}{\widehat{a}}_{i}}\dfrac{\sigma _{\widehat{a_{i}}} }{\sigma _{g_{i}}}g_{i}}{\sqrt{\sigma _{{\widehat{a}}_{i}}^{2}\left( 1-\dfrac{\sigma _{{\widehat{a}}_{i}}^{2}}{\sigma _{g_{i}}^{2}}\right) }}.$$

47 $$f_{sel}\left( {\mathbf {g}}_{sel}\right) = { \sum \limits _{i=1}^{m}} \log \left[ 1-\Phi \left( z_{i}\right) \right] .$$

The posterior distribution cannot be recognized and Markov chain Monte Carlo sampling may be considered for inference of ${\mathbf {g}}_{sel}$. A candidate-generating distribution in a Metropolis scheme [25, 45] could be

48 $${\mathbf {g}}_{sel}^{*}{|}\mathbf {y},\sigma _{a}^{2},\sigma _{e^{*}} ^{2}{\sim}N\left( \widetilde{{\mathbf {a}}}_{sel}{,}\left( {\mathbf {I}}_{sel}+\frac{\sigma _{e}^{2}}{\sigma _{a}^{2}}{\mathbf {A}} _{sel,sel}^{-1}\right) ^{-1}\sigma _{e}^{2}\right) ,$$

where $\widetilde{{\mathbf {a}}}_{sel}=\left( {\mathbf {I}}_{sel} +\frac{\sigma _{e}^{2}}{\sigma _{a}^{2}}{\mathbf {A}}_{sel,sel}^{-1}\right) ^{-1}{\mathbf {y}}_{sel}$. If ${\mathbf {g}}_{prop}^{*}$ is a draw from the proposal distribution, the probability of moving from state ${\mathbf {g}}^{now}$ to ${\mathbf {g}}_{prop}^{*}$ is $\alpha =\min \left[ R\left( {\mathbf {g}} ^{now},{\mathbf {g}}_{prop}^{*}\right) ,1\right]$, where

49 $$R\left( {\mathbf {g}}^{now},{\mathbf {g}}_{prop}^{*}\right) =\exp \left[ -\frac{1}{2\sigma _{e^{*}}^{2}}Q\left( {\mathbf {g}}^{now},{\mathbf {g}} _{prop}^{*}\right) +\Delta _{sel}\left( {\mathbf {g}}^{now},{\mathbf {g}} _{prop}^{*}\right) \right] ,$$

and

50 $$\begin{aligned}&Q\left( {\mathbf {g}}^{now},{\mathbf {g}}_{prop}^{*}\right) \\&\quad =\left( {\mathbf {g}}_{prop}^{*}-\widetilde{{\mathbf {g}}}_{sel}\right) ^{\prime }\widetilde{{\mathbf {C}}}_{g,sel}^{-1}\left( {\mathbf {g}}_{prop}^{*}-\widetilde{{\mathbf {g}}}_{sel}\right) -\left( {\mathbf {g}}^{now} -\widetilde{{\mathbf {g}}}_{sel}\right) ^{\prime }\widetilde{{\mathbf {C}}} _{g,sel}^{-1}\left( {\mathbf {g}}^{now}-\widetilde{{\mathbf {g}}}_{sel}\right) , \end{aligned}$$

51 $$\Delta_{sel} \left( {\mathbf{g}}^{now},\,{\mathbf{g}}_{prop}^{*}\right) = {\sum\limits _{i=1}^{m}} \log \frac{1-\Phi ( z_{i}^{now} ) }{1-\Phi \left( z_{i} ^{prop}\right) }.$$

The next state in the chain is given by the rule (*U* is an uniform random deviate):

52 $${\mathbf {g}}^{new}=\left\{ \begin{array}{c} {\mathbf {g}}_{prop}^{*}\text { if }U\le R\left( {\mathbf {g}}^{now} ,{\mathbf {g}}_{prop}^{*}\right) \\ {\mathbf {g}}^{now}\text { otherwise} \end{array} \right. .$$

**Fig. 3:**
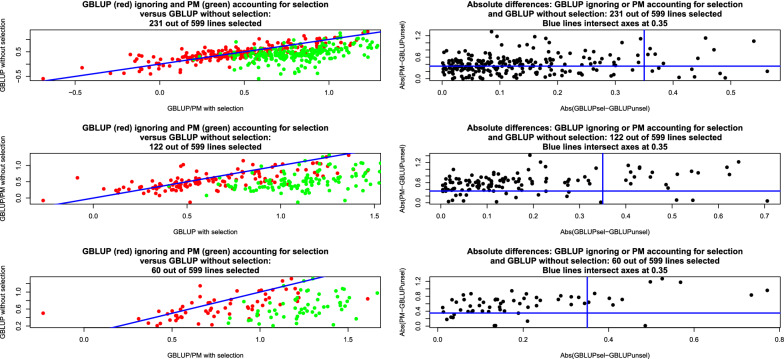
Posterior means without, ignoring or accounting for selection

**Fig. 4:**
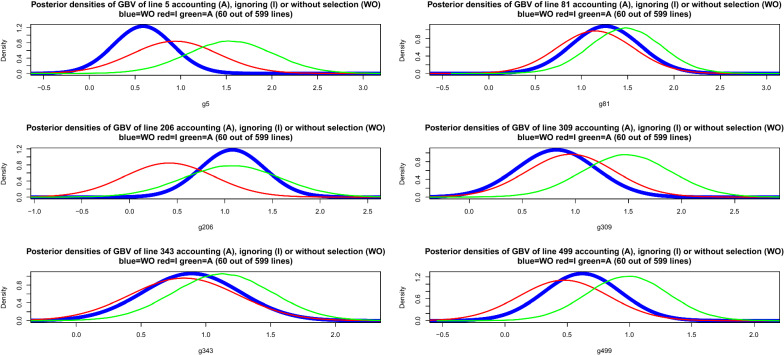
Posterior densities of six lines without, ignoring or accounting for selection

A Metropolis sampler with Eq. (48) as a proposal-generating process was used to estimate the posterior distribution having a density as given in Eq. (45) in scenarios where genotypes were available only for individuals whose $\widehat{{\mathbf {a}}}$ values exceeded 0.2,0.4 and 0.6, producing 231, 122 and 60 selected lines, respectively. The posterior distribution of genomic breeding values of the 599 lines prior to selection was ${\mathbf {g}}{|}\mathbf {y},\sigma _{g}^{2},\sigma _{e^{*}}^{2}{\sim}N\left( \widehat{{\mathbf {g}}},\mathbf {C}_{g}\right) ,$ as presented earlier. We also computed posterior distributions of the genomic breeding values ignoring the selection process from the data in selected lines. Metropolis sampling was done by running three chains (one per selection intensity) of length 100,000 each. After diagnostic tests [45, 46], a conservative burn-in period of 20,000 samples was adopted. Subsequently, a single long-chain of 480,000 iterations was run, with 380,000 samples used for inference, per setting. Figure 3 (left panels) depicts scatter plots of posterior means of genomic breeding values in the absence of selection (GBLUP without selection, y-axis) versus either GBLUP ignoring selection or posterior means accounting for selection (x-axis). Ignoring selection tended to overstate the estimates of genomic breeding values calculated without selection and from a larger sample ($n=599$ versus $n=231,122,60$ in the selection schemes). Accounting for selection produced estimated genomic breeding values (posterior means denoted as PM in the plots) that were even further away from those calculated without selection. The three panels at the right of Fig. 3 show larger differences between posterior means without selection (GBLUPunsel) and with selection accounted for (PM in the y-axis) than between GBLUPunsel and GBLUPsel, i.e., with the selection process ignored. Our way of accounting for selection produced larger absolute errors (taking GBLUPunsel as reference) than when selection was ignored, i.e., GBLUPsel. Posterior densities of the genomic breeding values of lines 5, 81, 206, 309, 343 and 499 are presented in Fig. 4; the data with selection pertain to the setting were 60 lines had been selected out of the 599 candidates. Densities ignoring selection (in red) tended to match better those obtained without selection (in blue) than the densities obtained with a selection model incorporated into inference (green). However, there was much uncertainty within each of the settings, leading to overlap, although the “green” density function was centered further right along the x-axis than the “blue” or “red” densities.

The following messages can be extracted from the example. First, paradoxically, our attempt at accounting for selection seemed to distort inference on the selected lines beyond what was obtained by ignoring selection: there was a noticeable overstatement of estimated breeding values. Second, it was not easy to account for selection even when the process leading to missing data was known. For instance, part of the information on selection had to be ignored because of the inability of writing the conditional distribution of genomic breeding values of unselected individuals, given those of the selected ones. That may be what “single-step” methods (e.g., [44]) implicitly do by including ungenotyped (but pedigreed) individuals in the analysis. Third, we employed variance parameters estimated prior to selection. This action was chosen because of the impossibility of obtaining sufficiently precise estimates given the small sizes of the selected samples of lines. Finally, at least in small samples it is always difficult to disentangle the impact of non-randomness from that of noise, in view of the uncertainty remaining after analysis, irrespective of whether selection is ignored or modelled explicitly.

### Example 3: multiple-trait sequential selection

A multiple-trait sequential selection scenario is described using two traits as an illustration, for simplicity. There is a set of *S* candidates (e.g., lines) with phenotypes $y_{11},y_{12},\ldots,y_{1S}$ available for trait 1, where the first subscript denotes the trait. A subset $S^{+}$ of the candidates is chosen to be measured for a second trait; the complementary subset $S^{-}$ contains candidates that have phenotypes for trait 1 but not for trait 2. The dataset presented for analysis contains only the pairs $\left\{ y_{1i},y_{2i}\right\}$ in $S^{+}$; pairs in $S^{-}$ are missing. Here, ${\mathbf{y}}_{obs}=\left( {\mathbf {y}}_{1S^{+}}^{\prime },{\mathbf {y}} _{2S^{+}}^{\prime }\right) ^{\prime }$ and ${\mathbf{y}}_{miss}=\left( {\mathbf {y}}_{1S^{-}}^{\prime },{\mathbf {y}}_{2S^{-}}^{\prime }\right) ^{\prime }$.

To define the distribution of the vector ${\mathbf {r}}$ describing the missing data pattern, an assumption about the selection process must be made. It will be assumed that a candidate is measured for trait 2 if its phenotype for trait 1 is larger than some ${{\varphi}}=y_{1,\min }$, a “minimum threshold of performance” for trait 1. Following [22], the conditional probability of selection (“fitness”) for candidate *i* is:

53 $$\Pr \left( r_{i}=1{{|}}{\mathbf{y}}_{obs}{,{\mathbf{y}} }_{miss}{,{\theta},}{{\varphi}}\right) =I_{\left( {{\varphi}} =y_{1,\min },\infty \right) }\left( y_{1i}\right) ;i=1,2,\ldots,S,$$

where ${\theta}$ are the unknown model parameters; $I_{\left( y_{1,\min },\infty \right) }\left( y_{1i}\right) =1$ if $y_{1i}>y_{1,\min }$ and 0 if $y_{1i}\le y_{1,\min }$. If, given ${\theta}$, pairs $\left\{ y_{1i},y_{2i}\right\}$ are mutually independent over individuals, the complete dataset and ${\mathbf {r}}$ have the joint density:

54 $$\begin{aligned}&p\left( {\mathbf{y}}_{obs},{\mathbf{y}}_{miss} ,{\mathbf {r}}|{{\varphi}}{,{\theta}}\right) \\&= { \prod \limits _{i\epsilon S^{+}}} p\left( y_{1i},y_{2i}|{\theta}\right) { \prod \limits _{i\epsilon S^{-}}} p\left( y_{1i},y_{2i}|{\theta}\right) I_{\left( -\infty ,{{\varphi}}\right) }\left( y_{1i}\right) \\&= { \prod \limits _{i\epsilon S^{+}}} p\left( y_{1i},y_{2i}|{\theta}\right) { \prod \limits _{i\epsilon S^{-}}} p\left( y_{2i}|y_{1i},{\theta}\right) p\left( y_{1i}|{\theta}\right) I_{\left( -\infty ,{{\varphi}}\right) }\left( y_{1i}\right) . \end{aligned}$$

Integrating out the missing data, i.e., $\left\{ y_{1i},y_{2i}\right\}$ in $S^{-}$, yields:

55 $$\begin{aligned}&p\left( {\mathbf{y}}_{obs},{\mathbf {r}}|{{{\varphi}},{\theta}}\right) \\&= { \prod \limits _{i\epsilon S^{+}}} p\left( y_{1i},y_{2i}|{\theta}\right) { \prod \limits _{i\epsilon S^{-}}} { \int } \left[ { \int } p\left( y_{2i}|y_{1i},{\theta}\right) dy_{2i}\right] I_{\left( -\infty ,{{\varphi}}\right) }\left( y_{1i}\right) p\left( y_{1i}|{\theta}\right) dy_{1i} \\&= { \prod \limits _{i\epsilon S^{+}}} p\left( y_{1i},y_{2i}|{\theta}\right) { \prod \limits _{i\epsilon S^{-}}} \Pr \left( y_{1i}<{{\varphi}}|{\theta}\right) . \end{aligned}$$

The posterior density is therefore:

56 $$\begin{aligned}&p\left( {\theta} {|} {\mathbf{y}}_{obs},\mathbf {r},Hyp\right) \\&\propto { \prod \limits _{i\epsilon S^{+}}} p\left( y_{1i},y_{2i}|{\theta}\right) p\left( {\theta}{|}Hyp\right) { \prod \limits _{i\epsilon S^{-}}} \Pr \left( y_{1i}<{{\varphi}}|{\theta}\right) \\&=\frac{p\left( {\theta} {|} {\mathbf{y}}_{obs},Hyp\right) \exp \left\{ { \sum \limits _{i\epsilon S^{-}}} \log \left[ \Pr \left( y_{1i}<{{\varphi}}|{\theta}\right) \right] \right\} }{ { \int } p\left( {\theta} {|} {\mathbf{y}}_{obs},Hyp\right) \exp \left\{ { \sum \limits _{i\epsilon S^{-}}} \log \left[ \Pr \left( y_{1i}<{{\varphi}}|{\theta}\right) \right] \right\} d{\theta}}. \end{aligned}$$

The wheat dataset was employed again to give a numerical illustration. The 599 lines have records in each of four distinct environments. To represent a scenario where selection does not occur, we fitted a four-variate linear model with the performances of the lines in different environments treated as distinct traits [34]. The model was:

57 $$\left( \begin{array}{c} {\mathbf {y}}_{1}\\ {\mathbf {y}}_{2}\\ {\mathbf {y}}_{3}\\ {\mathbf {y}}_{4} \end{array} \right) =\left( \begin{array}{c} {\mathbf {g}}_{1}\\ {\mathbf {g}}_{2}\\ {\mathbf {g}}_{3}\\ {\mathbf {g}}_{4} \end{array} \right) +\left( \begin{array}{c} {{\delta}}_{1}\\ {{\delta}}_{2}\\ {{\delta}}_{3}\\ {{\delta}}_{4} \end{array} \right) ,$$

where ${\mathbf {y}}_{i}$ $\left( i=1,2,3,4\right)$ is a $599\times 1$ vector of phenotypes for trait *i*,  the ${\mathbf {g}}_{i}^{\prime }s$ are genomic breeding values for the trait (marked with the 1279 markers) and ${{\delta}}_{i}$ is a model trait-specific residual vector. Prior assumptions were:

58 $$\begin{aligned} \left( \begin{array}{c} {\mathbf {g}}_{1}\\ {\mathbf {g}}_{2}\\ {\mathbf {g}}_{3}\\ {\mathbf {g}}_{4} \end{array} \right) |{\mathbf {G}}_{0}&\sim N\left( \left( \begin{array}{c} {\mathbf {0}}\\ {\mathbf {0}}\\ {\mathbf {0}}\\ {\mathbf {0}} \end{array} \right) ,{\mathbf {G}}_{0}\otimes {\mathbf {G}}\right) , \end{aligned}$$

59 $$\left( \begin{array}{c} {\mathbf {r}}_{1}\\ {\mathbf {r}}_{2}\\ {\mathbf {r}}_{3}\\ {\mathbf {r}}_{4} \end{array} \right) |{\mathbf {R}}_{0} \sim N\left( \left( \begin{array}{c} {\mathbf {0}}\\ {\mathbf {0}}\\ {\mathbf {0}}\\ {\mathbf {0}} \end{array} \right),{\mathbf {R}}_{0}\otimes {\mathbf {I}}_{599}\right),$$

where ${\mathbf {G}}$ is the genomic relationship matrix among the 599 lines; ${\mathbf {G}}_{0}$ is a $4\times 4$ between-trait genomic covariance matrix and ${\mathbf {R}}_{0}$ is a $4\times 4$ residual covariance matrix; residuals were independent between individuals, but a full covariance structure within individuals was posed. The four traits correspond to different locations so environmental correlations are expected to be null. However, we specified an unstructured ${\mathbf {R}}_{0}$ to account for correlations that are potentially created by non-additive genetic effects not accounted for in our additive genetic model, but known to exist for wheat yield. The two covariance matrices were estimated using a crude maximum likelihood procedure with ad-hoc adjustments to ensure positive-definiteness (a less crude but more involved algorithm would have given estimates that would not require any such adjustment). The estimates were subsequently taken as known and treated as hyper-parameters. The matrices (rounded values) were:

60 $${\mathbf {G}}_{0}=\left[ \begin{array}{cccc} 0.831 & -0.319 & -0.247 & -0.350\\ -0.319 & 0.750 & -0.195 & -0.213\\ -0.247 & -0.195 & 0.757 & -0.176\\ -0.350 & -0.213 & -0.176 & 0.752 \end{array} \right] ,$$

and

61 $${\mathbf {R}}_{0}=\left[ \begin{array}{cccc} 0.830 & -0.225 & -0.289 & -0.202\\ -0.225 & 0.872 & -0.330 & -0.317\\ -0.289 & -0.3330 & 0.918 & -0.352\\ -0.202 & -0.317 & -0.352 & 0.895 \end{array} \right] .$$

The matrix of phenotypic correlations between traits $\left( {\mathbf{{\Psi }}}\right)$, ${\mathbf {V}}_{0}={\mathbf {G}}_{0}+{\mathbf {R}}_{0},$ was:

$${\mathbf{{\Psi }}}=\left[ \begin{array}{cccc} 1 & -0.329 & -0.321 & -0.334\\ -0.329 & 1 & -0.3162 & -0.322\\ -0.321 & -0.3162 & 1 & -0.318\\ -0.334 & -0.322 & -0.318 & 1 \end{array} \right] .$$

The adjustment for positive-definiteness produced estimates of phenotypic correlations (negative and similar between pairs of traits). Traits turned out to be negatively correlated at genetic, residual and phenotypic levels. The correlation values should not be interpreted inferentially as the adjustment was done solely to facilitate calculation and for illustrative purposes.

Prior to selection, each line had a grain yield in each environment. The data available for analysis post-selection (phenotypes centered and standardized) included the lines exceeding the performance thresholds $t_{1}=0$, $t_{2}=0.6$ and $t_{3}=1.3$ units above the mean in environments 1, 2 and 3, respectively, so only lines with performance above the minimum values for the three traits had records in environment 4. Only 10 such lines met the “culling levels”, so phenotypes presented to the hypothetical analyst consisted of a $10\times 4$ matrix, lines in rows and traits in columns. Ignoring selection, the posterior distribution (given ${\mathbf {G}}_{0}$ and ${\mathbf {R}}_{0}$) of the genomic breeding values using the selected dataset is multivariate normal, with the mean vector:

62 $$\widetilde{{\mathbf {g}}}_{sel}=\left( \begin{array}{c} \widetilde{{\mathbf {g}}}_{1,sel}\\ \widetilde{{\mathbf {g}}}_{2,sel}\\ \widetilde{{\mathbf {g}}}_{3,sel}\\ \widetilde{{\mathbf {g}}}_{4,sel} \end{array} \right) =\left( {\mathbf {G}}_{0}\otimes {\mathbf {G}}_{sel,sel}\right) \left( {\mathbf {G}}_{0}\otimes {\mathbf {G}}_{sel,sel}+{\mathbf {R}}_{0}\otimes {\mathbf {I}} _{10}\right) ^{-1}\left( \begin{array}{c} {\mathbf {y}}_{1,sel}\\ {\mathbf {y}}_{2,sel}\\ {\mathbf {y}}_{3,sel}\\ {\mathbf {y}}_{4,sel} \end{array} \right) ,$$

and covariance matrix

63 $${\mathbf {C}}_{g,sel}=\left( {\mathbf {G}}_{0}\otimes {\mathbf {G}}_{sel,sel}\right) \left[ {\mathbf {I}}-\left( {\mathbf {G}}_{0}\otimes {\mathbf {G}}_{sel,sel} +{\mathbf {R}}_{0}\otimes {\mathbf {I}}_{10}\right) ^{-1}\left( {\mathbf {G}} _{0}\otimes {\mathbf {G}}_{sel,sel}\right) \right] .$$

Above, $\widetilde{{\mathbf {g}}}_{i,sel}$ is a $10\times 1$ vector of posterior means of genomic breeding values in the ten selected lines for trait *i*;  ${\mathbf {G}}_{sel,sel}$ is the $10\times 10$ genomic relationship matrix between selected lines, and ${\mathbf {y}}_{i,sel}$ is their vector of phenotypes.

Under the multivariate selection scheme adopted, Eq. (56) is expressible as:

64 $$\begin{aligned} & p\left({\theta {|}} {\mathbf {y}}_{obs},\mathbf {r}, Hyp\right) \\ &\propto { \prod \limits _{i\epsilon S^{+}}} p\left( y_{1i},y_{2i},y_{3i},y_{4i}|{\theta}\right) p\left({\theta {|}}Hyp\right) { \prod \limits _{i\epsilon S^{-}}} \Pr \left( y_{1i}<t_{1},y_{2i}<t_{2},y_{3i}<t_{3}|{\theta}\right) \\& \propto { \prod \limits _{i\epsilon S^{+}}} p\left( y_{1i},y_{2i},y_{3i},y_{4i}|{\theta}_{sel}\right) p\left( {\theta}_{sel}{|}Hyp\right) \\ & \times { \prod \limits _{i\epsilon S^{-}}} \Pr \left( y_{1i}<t_{1},y_{2i}<t_{2},y_{3i}<t_{3}|{\theta} _{nsel}\right) p\left({\theta}_{nsel}|{\theta}_{sel} ,Hyp\right) \\ & \propto p\left( {\theta}_{sel}{|}{\mathbf {y}}_{obs},Hyp\right) { \prod \limits_{i\epsilon S^{-}}} \Pr \left( y_{1i}<t_{1},y_{2i}<t_{2},y_{3i}<t_{3}|{\theta} _{nsel}\right) p\left({\theta}_{nsel}|{\theta}_{sel} ,Hyp\right) . \end{aligned}$$

Above,

65 $$p\left( {\theta}_{sel}{|}{\mathbf{y}}_{obs},Hyp\right) \propto \exp \left[ -\frac{1}{2}\left( {\mathbf {g}}_{sel}-\widetilde{{\mathbf {g}}}_{sel}\right) ^{\prime }{\mathbf {C}}_{g,sel}^{-1}\left( {\mathbf {g}}_{sel}-\widetilde{{\mathbf {g}} }_{sel}\right) \right] ,$$

66 $$\begin{aligned}&\Pr \left( y_{1i}<t_{1},y_{2i}<t_{2},y_{3i}<t_{3}|{\theta} _{nsel}\right) \\&= { \int \limits _{-\infty }^{t_{1}}} { \int \limits _{-\infty }^{t_{2}}} { \int \limits _{-\infty }^{t_{3}}} \phi \left( {\mathbf {y}}_{i,nsel}|{\mathbf {g}}_{i,nsel},\left[ \begin{array}{ccc} R_{11} & R_{12} & R_{13}\\ R_{21} & R_{22} & R_{23}\\ R_{31} & R_{32} & R_{33} \end{array} \right] ,t_{1},t_{2},t_{3}\right) d{\mathbf {y}}_{i,nsel} \\&=\Phi \left( {\mathbf {y}}_{i,nsel}|{\mathbf {g}}_{i,nsel},{\mathbf {R}}_{0} ,t_{1},t_{2},t_{3}\right) ;i\epsilon S^{-}, \end{aligned}$$

where $\phi \left( .\right)$ is the density of a trivariate normal distribution with the mean vector ${\mathbf {g}}_{i,nsel}=\left( g_{1i,nsel},g_{2i,nsel},g_{2i,nsel}\right) ^{\prime }$, $R_{ij}$ is an appropriate element of ${\mathbf {R}}_{0},$ and $\Phi \left( .\right)$ is a trivariate normal distribution function. Further, $p\left( {\theta}_{nsel}|{\theta}_{sel},Hyp\right)$ is the density of a multivariate normal distribution with mean vector

67 $${\mathbf {m}}_{n|s}=\left( {\mathbf {G}}_{0}\otimes {\mathbf {G}}_{nsel,sel}\right) \left( {\mathbf {G}}_{0}^{-1}\otimes {\mathbf {G}}_{sel,sel}^{-1}\right) {\mathbf {g}}_{sel}=\left( {\mathbf {I}}_{4}\otimes {\mathbf {G}}_{nsel,sel} {\mathbf {G}}_{sel,sel}^{-1}\right) {\mathbf {g}}_{sel},$$

and covariance matrix

68 $${\mathbf {V}}_{n|s}={\mathbf {G}}_{0}\otimes {\mathbf {G}}_{nsel,nsel}-{\mathbf {G}} _{0}\otimes {\mathbf {G}}_{nsel,sel}{\mathbf {G}}_{sel,sel}^{-1}{\mathbf {G}}_{sel,nsel}$$

Collecting terms,

69 $$p\left( {\theta} {|} {\mathbf{y}}_{obs},\mathbf {r},Hyp\right) \propto \exp \left[ -\frac{1}{2}\left( Q_{sel}+Q_{nsel}\right) +\Delta \left( {\mathbf {y}} _{nsel},{\mathbf {g}}_{nsel}\right) \right]$$

where

70 $$\begin{aligned} Q_{sel}= & \left( {\mathbf {g}}_{sel}-\widetilde{{\mathbf {g}}}_{sel}\right) ^{\prime }{\mathbf {C}}_{g,sel}^{-1}\left( {\mathbf {g}}_{sel}-\widetilde{{\mathbf {g}} }_{sel}\right) , \end{aligned}$$

71 $$\begin{aligned} Q_{nsel}= & \left( {\mathbf {g}}_{nsel}-{\mathbf {m}}_{n|s}\right) ^{\prime }{\mathbf {V}}_{n|s}^{-1}\left( {\mathbf {g}}_{nsel}-{\mathbf {m}}_{n|s}\right) , \end{aligned}$$

72 $$\begin{aligned} \Delta \left( {\mathbf {y}}_{nsel},{\mathbf {g}}_{nsel}\right)= & { \sum \limits _{i\epsilon S^{-}}} \log \left[ \Phi \left( {\mathbf {y}}_{i,nsel}|{\mathbf {g}}_{i,nsel},{\mathbf {R}} _{0},t_{1},t_{2},t_{3}\right) \right] . \end{aligned}$$

To estimate the posterior distribution, a Metropolis algorithm was tailored as in Example 2. Here, the proposal distribution (with dimension 599) was a multivariate normal distribution with mean vector equal to:

73 $$\left[ \begin{array}{c} {{{\mu }}}_{1}\\ {{{\mu }}}_{2}\\ {{{\mu }}}_{3}\\ {{{\mu }}}_{4} \end{array} \right] =\left[ \begin{array}{c} G_{11}{\mathbf {A}}\left( G_{11}\mathbf {A+}2R_{11}{\mathbf {I}}_{599}\right) ^{-1}{\mathbf {y}}_{1}\\ G_{22}{\mathbf {A}}\left( G_{22}\mathbf {A+}2R_{22}{\mathbf {I}}_{599}\right) ^{-1}{\mathbf {y}}_{2}\\ G_{33}{\mathbf {A}}\left( G_{33}\mathbf {A+}2R_{33}{\mathbf {I}}_{599}\right) ^{-1}{\mathbf {y}}_{3}\\ G_{44}{\mathbf {A}}\left( G_{44}\mathbf {A+}2R_{44}{\mathbf {I}}_{599}\right) ^{-1}{\mathbf {y}}_{4} \end{array} \right] {,}$$

and block-diagonal covariance matrix

74 $${\mathbf {D}}={\oplus}_{i=1}^{4}G_{ii}{\mathbf {A}}\left[ {\mathbf {I}}_{599} - \left( G_{ii}\mathbf {A+}2R_{ii}{\mathbf {I}}_{599}\right) ^{-1} G_{ii}{\mathbf {A}}\right] .$$

**Fig. 5:**
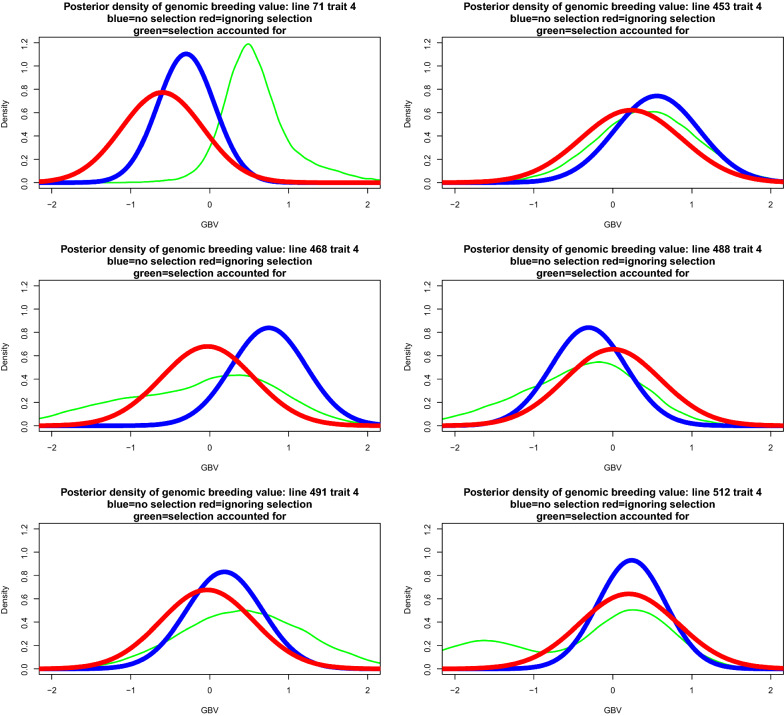
Posterior densities of six lines without, ignoring or accounting for selection: tetra-variate analysis

The proposal distribution used an overdispersed covariance matrix. We ran four separate chains: two had 2500 iterations each, and the third and fourth ones had 5000 and 40,000 iterations, respectively. The *R* metric [46] indicated that convergence had been attained at about iteration 1500. Conservatively, the last 500 iterations of chains 1 and 2 were saved for inference; likewise, 3000 and 38,000 iterations were saved from chains 3 and 4, respectively. Posterior distributions of the genomic breeding values of the 10 selected lines were estimated from the 42,000 samples available for inference. Figure 5 displays posterior densities of 6 out of the 10 selected lines. Ideally, we would expect the blue and green curves to separate from the red curve (ignoring selection). Because selection was so severe (10 out of an initial 599), the three Bayesian analyses displayed great uncertainty, especially the one where the selection process was accounted for. This example suggests that, even when the selection or dropout process is known, accounting for it may not be a fruitful process.

### Example 4: nor-optimal selection is not ignorable

Often, selection is of a stabilizing form and aimed at moving the population towards some optimum value. A model for such selection was introduced by [26] and used later by [29, 47]. Let $${\mathbf {y}} =\left[ \begin{array}{cc} {\mathbf {y}}_{1}^{\prime },&{\mathbf {y}}_{2}^{\prime } \end{array} \right] ^{\prime }$$be a vector of random variables; some of its components may be unobservable. Without selection, assume:

75 $$\left[ \begin{array}{c} {\mathbf {y}}_{1}\\ {\mathbf {y}}_{2} \end{array} \right] \sim N \left({\mathbf {m}}=\left[ \begin{array}{c} {\mathbf {m}}_{1}\\ {\mathbf {m}}_{2} \end{array} \right] ,{\mathbf {V}}=\left[ \begin{array}{cc} {\mathbf {V}}_{11} & {\mathbf {V}}_{12}\\ {\mathbf {V}}_{21} & {\mathbf {V}}_{22} \end{array} \right]\right),$$

where the ${\mathbf {m}}^{\prime }$s are mean vectors and the ${\mathbf {V}}^{\prime }$s are covariance matrices. Selection operates on ${\mathbf {y}}_{1}$ through the Gaussian fitness function

76 $$H({\mathbf {y}}_{1})=\exp \left[-\frac{1}{2}({\mathbf {y}}_{1}-{\lambda})^{\prime }{\boldsymbol{\Gamma}}^{-1}({\mathbf {y}}_{1}-{\lambda})\right],$$

where ${\lambda}$ is a vector-valued “optimum” and the positive-definite matrix $\mathbf{\Gamma}$ describes the sharpness of multivariate selection. The fitness function is symmetric about ${\lambda}$ and has a maximum value of 1 when ${\mathbf {y}}_{1}={\lambda}$; values of ${\mathbf {y}}_{1}$ “far away” from ${\lambda}$ confer lower fitness. In a single-variable situation, fitness takes the form $H\left( y\right) =\exp [-\dfrac{\left( y-\lambda \right) ^{2}}{2\gamma }]$. A smaller $\gamma$ implies a sharper decay in fitness when *y* deviates from $\lambda$; larger values denote a more gentle selection, with no selection at all if $\gamma =\infty$. Write

77 $$H({\mathbf {y}}_{1})=\exp \left[-\frac{1}{2}({\mathbf {y}}-{\lambda}_{0})^{\prime }{\boldsymbol{\Gamma}}_{0}^{-}({\mathbf {y}}-{\lambda}_{0})\right],$$

where $${\lambda}_{0}^{\prime }=\left[ \begin{array}{cc} {\lambda}^{\prime }&{\mathbf {0}} \end{array} \right]$$; ${\mathbf {0}}$ is a null vector with order equal to that of ${\mathbf {y}}_{2}$ and $${\boldsymbol{\Gamma}}_{0}^{-}=\left[ \begin{array}{cc} {\boldsymbol{\Gamma}}^{-1} & {\mathbf {0}}\\ {\mathbf {0}} & {\mathbf {0}} \end{array} \right]$$. The density of ${\mathbf {y}}$ after selection acting on ${\mathbf {y}}_{1}$ (ignoring dependence on parameters in the notation) is:

78 $$p_{s}({\mathbf {y}}_{1},{\mathbf {y}}_{2})=\frac{H({\mathbf {y}}_{1})p({\mathbf {y}} _{1},{\mathbf {y}}_{2})}{\iint H({\mathbf {y}}_{1})p({\mathbf {y}}_{1},{\mathbf {y}} _{2})d{\mathbf {y}}_{1}d{\mathbf {y}}_{2}}=\frac{H({\mathbf {y}}_{1})}{\overline{H} }p({\mathbf {y}}_{1},{\mathbf {y}}_{2});$$

equivalently, the density in “survivors” is:

79 $$p_{s}({\mathbf {y}}_{1},{\mathbf {y}}_{2})\propto H({\mathbf {y}}_{1})p({\mathbf {y}} _{1},{\mathbf {y}}_{2}).$$

Using Eq. (77), the post-selection density becomes:

80 $$p_{s}({\mathbf {y}}_{1},{\mathbf {y}}_{2})\propto \exp \left\{-\frac{1}{2} \left[{({\mathbf {y}}-{\lambda}}_{0})^{\prime}{\boldsymbol{\Gamma}}_{0}^{-} ({\mathbf {y}}-{\lambda}_{0})+({\mathbf {y}}-{\mathbf {m}})^{\prime }{\mathbf {V}} ^{-1}({\mathbf {y}}-{\mathbf {m}})\right]\right\}.$$

Combining the two quadratic forms on ${\mathbf {y}}$ in Eq. (80) yields

81 $$\begin{aligned}&\left( {\mathbf {y}}-{\lambda}_{0}\right) ^{\prime}{\boldsymbol{\Gamma}}_{0}^{-}\left( {\mathbf {y}}-{\lambda}_{0}\right) +\left( {\mathbf {y}}-{\mathbf {m}}\right) ^{\prime }{\mathbf {V}}^{-1}\left( {\mathbf {y}}-{\mathbf {m}}\right) \\&\quad = \left( {\mathbf {y}}-{\mathbf {m}}_{s}\right) ^{\prime }\left({\boldsymbol{\Gamma}}_{0} ^{-}+{\mathbf {V}}^{-1}\right) \left( {\mathbf {y}}-{\mathbf {m}}_{s}\right) {+({\lambda}}_{0}-\mathbf {m)}^{\prime}{\boldsymbol{\Gamma}}_{0}^{-}\left({\boldsymbol{\Gamma}}_{0}^{-}+{\mathbf {V}}^{-1}\right) ^{-1}{\mathbf {V}}^{-1} {({\lambda}}_{0}-\mathbf {m)} \end{aligned}$$

Above,

82 $${\mathbf {m}}_{s}=\left({\boldsymbol{\Gamma}}_{0}^{-}+{\mathbf {V}}^{-1}\right) ^{-1}({\boldsymbol{\Gamma}}_{0}^{-}{\lambda}_{0}+{\mathbf {V}}^{-1}{\mathbf {m}})$$

Since the second component of Eq. (81) does not involve ${\mathbf {y}}$, (80) can be written as:

83 $$p_{s}({\mathbf {y}}_{1},{\mathbf {y}}_{2})\propto exp\left\{-\frac{1}{2}\left( {\mathbf {y}}-{\mathbf {m}}_{S}\right) ^{\prime }\left({\boldsymbol{\Gamma}}_{0}^{-} +{\mathbf {V}}^{-1}\right) \left( {\mathbf {y}}-{\mathbf {m}}_{S}\right) \right\}.$$

Hence, the joint distribution of ${\mathbf {y}}$ remains normal in survivors to selection but with different parameters. Therefore, the post-selection distribution is:

84 $$[{\mathbf {y}}]_{s}\sim N[{\mathbf {m}}_{s},{\mathbf {V}}_{s}=({\boldsymbol{\Gamma}}_{0}^{-}+{\mathbf {V}}^{-1})^{-1}].$$

Note that

85 $${\mathbf {V}}_{s}=({\boldsymbol{\Gamma}}_{0}^{-}+{\mathbf {V}}^{-1})^{-1}={\mathbf {V}} ({\mathbf {I}}+{\boldsymbol{\Gamma}}_{0}^{-}{\mathbf {V}})^{-1}$$

where $({\mathbf {I}}+{\boldsymbol{\Gamma}}_{0}^{-}{\mathbf {V}})^{-1}$ is related to the “coefficient of centripetal selection” *S* [29]. In a scalar situation $({\mathbf {I}}+{\boldsymbol{\Gamma}}_{0} ^{-}{\mathbf {V}})^{-1}=\dfrac{\gamma }{V+\gamma }=1-S,$ gives the fraction of variance $\left( V\right)$ remaining after selection and $S=\dfrac{V}{V+\gamma }$ measures the fraction removed by nor-optimal selection.

We use a canonical setting to show that selection is not ignorable, i.e., selection must be modelled for appropriate inference. Assume that the mean and the additive genetic and environmental variance components prior to selection are known. The setting is $y=u+e,$ where $u\sim N(0,h^{2})$ and $e\sim N(0,1-h^{2})$ are independently distributed standardized breeding values and environmental effects, respectively, and $h^{2}$ is trait heritability. The marginal distribution of phenotypes is $y\sim N(0,1).$ Without selection,

86 $$\left[ \begin{array}{c} y\\ u \end{array} \right] \sim N\left( \left[ \begin{array}{c} 0\\ 0 \end{array} \right] ,\left[ \begin{array}{cc} 1 & h^{2}\\ h^{2} & h^{2} \end{array} \right] \right),$$

$E(u|y)=h^{2}y$, and $Var(u|y)=h^{2}(1-h^{2}).$ After a round of phenotypic selection towards $\lambda$ with sharpness $\gamma$, the joint distribution of breeding values and phenotypes remains bivariate normal. Post-selection,

87 $$E_{s}\left[ \begin{array}{c} y\\ u \end{array} \right] =\left[ \begin{array}{c} \lambda S\\ h^{2}\lambda S \end{array} \right] ,$$

and

88 $$Var_{s}\left[ \begin{array}{c} y\\ u \end{array} \right] =\left[ \begin{array}{cc} 1-S & h^{2}\left( 1-S\right) \\ h^{2}\left( 1-S\right) & h^{2}(1-h^{2}S) \end{array} \right] ,$$

respectively, where $S=\dfrac{1}{1+\gamma }$. The additive variance is a fraction $1-h^{2}S$ of the genetic variation prior to selection. Post-selection, the best predictor [42, 48] of *u* is:

89 $$E_{s}\left( u|y\right) =h^{2}\lambda S+\frac{h^{2}\left( 1-S\right) }{1-S}\left( y-\lambda S\right) =h^{2}y,$$

and

90 $$Var_{s}\left( u|y\right) =h^{2}(1-h^{2}S)-\frac{h^{4}\left( 1-S\right) ^{2}}{1-S}=h^{2}\left( 1-h^{2}\right) .$$

The parameters of the conditional distribution $\left[ u|y\right]$ are as prior to selection, so the latter is ignorable from the point of view of learning *u* from *y*.

Suppose now that the model is $y_{i}=\mu +u_{i}+e_{i}$ with $u\sim N(0,h^{2})$ and $e\sim N(0,1-h^{2})$ as before; $h^{2}$ is known but $\mu$ is unknown. Given a sample of size *n*,  without selection, the posterior distribution of $\mu$ after assigning a flat prior to the latter parameter is $\mu |{\mathbf {y}}{\sim}N\left( \overline{y},n^{-1}\right)$ where $\overline{y}=\dfrac{{\sum \nolimits _{i=1}^{n}} y_{i}}{n}$ is the maximum likelihood estimator of $\mu$ [25]. The analyst, however, is presented with a sample of *m* individuals known to belong to a population subjected to stabilizing selection towards $\lambda$ with coefficient of selection *S*. After selection, the marginal distribution of the phenotypes is:

91 $$N\left( \mu _{s}=\mu \left( 1-S\right) +\lambda S,V_{s}=\left( 1-S\right) \right) .$$

If a flat prior is assigned to $\mu _{s},$ then $\mu _{s}|{\mathbf {y}}{\sim}N\left( \dfrac{{ \sum \nolimits _{i=1}^{m}} y_{i}}{m},(1-S)m^{-1}\right)$. What is the posterior distribution of $\mu$? Changing variables $\mu _{s}\longrightarrow \mu$,

$$\begin{aligned} p_{s}\left( \mu |{\mathbf {y}}\right)&\propto \left( 1-S\right) p_{s}\left( \mu _{s}|{\mathbf {y}}\right) \\&\propto \exp \left\{ -\frac{m}{2(1-S)}\left[ \mu \left( 1-S\right) +\lambda S-\dfrac{ { \sum \limits _{i=1}^{m}} y_{i}}{m}\right] ^{2}\right\} \\&\propto \exp \left\{ -\frac{m(1-S)}{2}\left[ \mu -\frac{\overline{y}-\lambda S}{1-S}\right] ^{2}\right\} . \end{aligned}$$

The preceding implies that the posterior distribution of $\mu$ after selection changes to $\left[ \mu |{\mathbf {y}}\right] _{s}\sim N\left[ \dfrac{\overline{y}-\lambda S}{1-S},\dfrac{1}{n(1-S)}\right]$. In short, the missing data process must be considered from the point of view of inferring the mean of the base population, but can be ignored for learning the additive genetic value *u*.

## Discussion

Selection is a central theme in evolutionary and applied quantitative genetics [12]. Yet, textbooks and papers place focus on stylized models, with less emphasis on parameter inference using data from real selection processes. The literature from animal breeding has special relevance because their data derive mostly from farm records of performance with incomplete reporting and follow-up, especially of events leading to non-randomly missing observations. In this section, some landmark papers on the topic are discussed and their messages are contrasted with our results.

A large part of the animal breeding literature in the first six or seven decades of the 20th century reported randomized selection experiments, typically possessing a small scale and insufficient resolution or replication [49]. By virtue of design, the analysis of these experiments was not too challenging, statistically speaking, and much work centered on the assessment of the expected variability of response to selection in unreplicated experiments [50]. Perhaps the first formal attempt at addressing distortion in inference from observational data generated by a type of culling employed in animal breeding was [30]. Suppose ${\mathbf {y}}_{0},{\mathbf {y}}_{1}\in S_{1}$ and ${\mathbf {y}}_{2}\in S_{2}$ are observable data derived from a sequential selection of individuals, i.e., ${\mathbf {y}}_{0}\rightarrow {\mathbf {y}}_{1}\longrightarrow {\mathbf {y}}_{2}$ where $S_{1}$ and $S_{2}$ are sampling spaces modified by selection, while ${\mathbf {y}}_{0}$ has unrestricted space. Based on ${\mathbf {y}}_{0}$, then ${\mathbf {y}}_{1}$ is observed, and given ${\mathbf {y}}_{0}$ and ${\mathbf {y}}_{1}$, data ${\mathbf {y}}_{2}$ are collected. These authors showed bias of linear ordinary least squares estimators of production differences between, e.g., ages of cow or of time trends when there was sequential selection. The least squares estimators examined were: (a) difference between averages of second lactation records (${\mathbf {y}}_{1}$) and of all first records ${\mathbf {y}}_{0}$ (gross comparison), and (b) between second and first lactation averages, but only for cows that had the two records of production (paired comparison). In the gross comparison, missing data for second records were those for cows with lower “fitness” due to having lower first production records. In the paired comparison, the missing data were all records (first and second lactation) of cows not given an opportunity to have a second lactation. In the absence of selection, a multivariate normal joint distribution was assumed by [30], with the fixed parameter vector ${\theta}$ (fixed effects and variance-covariance components) inferred by maximum likelihood. Henderson [30] noted that if all available records are used in the analysis, the maximum likelihood estimator of age effects, i.e., a location parameter, would not contain bias even if selection is ignored in the analysis. Under normality and with a general but known covariance structure, the maximum likelihood estimator is generalized least squares, not ordinary least squares. Their paper did not address inference of unobserved producing abilities or of random genetic effects such as breeding values, which are factors underpinning the non-diagonal covariance matrix structure.

More generally, let $\left[ {\mathbf {y}}_{0},{\mathbf {y}}_{1},{\mathbf {y}} _{2};{\theta}\right]$ represent the joint distribution (does not need to be Gaussian) of the ${\mathbf {y}}$ vectors, indexed by some parameter ${\theta}$. Apart from numerical issues, the maximum likelihood estimates are straightforward to obtain because of the automaticity of the method. Since the conditional distributions $\left[ {\mathbf {y}}_{1} |{\mathbf {y}}_{0};{\theta}\right]$ and $\left[ {\mathbf {y}} _{2}|{\mathbf {y}}_{0},{\mathbf {y}}_{1};{\theta}\right]$ hold for any ${\mathbf {y}}_{0}$ and ${\mathbf {y}}_{1}$, the joint density can be written as $p\left( {\mathbf {y}}_{0}|{\theta}\right) p\left( {\mathbf {y}}_{1}|{\mathbf {y}}_{0};{\theta}\right) p\left( {\mathbf {y}} _{2}|{\mathbf {y}}_{0},{\mathbf {y}}_{1};{\theta}\right) =p\left( {\mathbf {y}}_{0},{\mathbf {y}}_{1},{\mathbf {y}}_{2};{\theta}\right)$. Hence, the likelihood function (any part of the joint density involving ${\theta}$) is unaffected by selection and the non-random process can be ignored when computing maximum likelihood estimates of ${\theta}$ if such sequential mechanism takes place. However, the asymptotic properties of the estimators are affected by selection, since Fisher’s expected information must be computed by taking expectations with respect to $\left[ {\mathbf {y}}_{0},{\mathbf {y}}_{1},{\mathbf {y}}_{2};\mathbf {\theta ,y}_{1}\in S_{1},{\mathbf {y}}_{2}\in S_{2}\right]$ instead of $\left[ {\mathbf {y}} _{0},{\mathbf {y}}_{1},{\mathbf {y}}_{2};{\theta}\right]$; a selection model must be adopted for calculation of expected second derivatives. This was done by [51] for estimation of repeatability of production in dairy cattle.

Maximum likelihood estimates are typically biased in finite samples even without selection, e.g., the maximum likelihood estimator of residual variance in linear regression models has a downwards bias. In an early study, Rothschild et al. [52] examined bias of estimates of genetic parameters (based on variance and covariance components) by simulating first and second lactation records in dairy cattle. The selection scheme was stylized: 50 % of 5000 progeny of 100 bulls was allowed to have second records, and individuals were chosen either at random or were those with the highest first record of production; the scheme was replicated 200 times. The simulation did not detect bias of estimates of heritability of first and second records, or of genetic and phenotypic correlations between the two lactations. However, the mean squared error of the estimates was larger under selection than under a random choice, illustrating that the finite sample distribution of maximum likelihood estimates is affected by selection. In other words, ignorability of selection cannot be claimed without qualification: it does not necessarily imply bias removal or unaffected sampling distribution of estimates. Ignorability means that the likelihood function can be constructed as if selection had not taken place.

Results in [30] carry beyond normality because composition of a joint density as a sequence of conditional densities follows directly from probability theory. However, selection is often based on unobserved “externalities” as well as on data available for analysis, as discussed in our paper. For instance, in a clinical trial, a patient may abandon the study due to some unrecorded event, e.g., tooth ache, that may be treatment-related, so some component of ${\theta}$ affects a missing process involving unobserved data. The notion of ignorability of missingness of data was formalized in [21] and adapted to likelihood-based inference in animal breeding by Im et al. [22]. The main messages of such work are: if the probability of missingness (our fitness function is an equivalent metric) depends on observed data only, or if it involves parameters that are “distinct” from ${\theta}{,}$ the selection process can be ignored from the point of view of locating the maximizer of the likelihood.

Apart from genetic and environmental parameters, animal and plant breeders also seek estimates of unobservable breeding values, i.e., quantities that vary over individuals and that are not construed as parameters in classical inference. However, results in [30] do not apply without qualification to inference of unobservable (but realized) random variables (called “prediction” of a random vector ${\mathbf {u}}$), since estimation and prediction are treated distinctly in frequency and likelihood-based approaches. Actually, the mixed model equations algorithm was derived in their paper by joint maximization with respect to fixed effects and $\mathbf {u},$ of a “penalized” likelihood function under Gaussian assumptions and known dispersion structure, incorrectly interpreted as a classical likelihood. The ${\mathbf {u}}$-solution of the estimating equations was later shown to correspond to the BLUP, and for many years was wrongly referred to as “maximum likelihood estimator” of ${\mathbf {u}}$**.** Since the the penalized likelihood is proportional $\left[ {\mathbf {y}}_{0},{\mathbf {y}}_{1} ,{\mathbf {y}}_{2},{\mathbf {u}};{\theta}\right]$ one can also argue that the penalized maximum likelihood estimator of ${\mathbf {u}}$ (that is, what we would call BLUP) could be calculated ignoring the sequential selection process ${\mathbf {y}}_{0}\rightarrow {\mathbf {y}}_{1}\longrightarrow {\mathbf {y}}_{2}.$

Perhaps the bias issue is what motivated Henderson et al. [30] to study BLUP under the form of selection described by Pearson [53], who had shown how selection operating upon a multivariate normal distribution altered its mean vector and covariance matrix. A paper [54] noted that a special case of Pearson–Henderson selection is the truncation scheme in textbooks of quantitative genetics [55]. Under Pearson’s model and Gaussian assumptions, the first and second moments of the joint distribution of a set of random variables (observed or latent) after selection, can be arrived at analytically; formulae apply to a single cycle of selection only, as multivariate normality is destroyed post-selection. Assuming the dispersion structure was known [43], derived conditions under which Pearsonian selection could be ignored in the computation of BLUP. Essentially, he considered linear predictors, ${\mathbf{L}}^{\prime} {\mathbf {y}}$ of an unobservable random vector ${\mathbf {u}}$, such that $E_{s}\left({\mathbf {L}}^{\prime} {\mathbf {y}}\right) =E_{s}\left( {\mathbf {u}}\right) ,$ where *s* denotes Pearsonian selection. In this class of predictors, Henderson [43] searched for the ${\mathbf {L}}$ matrix that produced minimum variance of prediction error. Two of his results have had a marked influence on animal breeding modeling. One was that, if $E\left( {\mathbf {L}}^{\prime} {\mathbf {y}}\right) ={\mathbf {0}}$ (location invariance), the selection process could be ignored, with BLUP computed as if selection had not taken place. The other one, was that if selection had been based on the linear combination ${\mathbf {L}}^{\prime}{\mathbf {u}}$, by treating fixed effects levels of other random vectors in the model (e.g., contemporary groups) as fixed effects one could arrive at unbiased predictors of ${\mathbf {u}}.$

Henderson’s treatment of selection was discussed critically by, e.g., [11] and by [56, 57]. The frequentist setting in [43] assumed that the ${\mathbf {L}}$ matrix was constant (and, therefore, the incidence matrices in the linear model) over conceptual repeated sampling. This assumption is not reasonable, as a conceptual replication with the same distribution of observations over subclasses could not be expected to occur with unbalanced field data collected over a large number of contemporary groups and several years. The most widely cited result was that BLUP is unaffected by selection if the criterion used for ranking (${\mathbf {L}}^{\prime} {\mathbf {y}}$) has a probability distribution that is translation invariant. This requirement is violated in animal breeding any time that a model with fixed “genetic group effects” is used, a routine modeling strategy, e.g., in beef cattle evaluation; in this case ${\mathbf {L}}^{\prime }{\mathbf {y}}$ has a non-null expectation. The ${\mathbf {L}}^{\prime }{\mathbf {u}}$ development is logically difficult to follow. For instance, selection based on ${\mathbf {L}} ^{\prime }{\mathbf {u}}$ requires knowledge of ${\mathbf {u}}$, so there, predicting this latter vector would not be needed. The study of Schaeffer [58] discussed pros and cons of treating a large number of contemporary groups as fixed effects, the strategy recommended by Henderson to eliminate biases due to non-random associations between breeding values of bulls (used over many herds) and farm effects, which the latter interpreted as a special case of selection based on ${\mathbf {L}}^{\prime }{\mathbf {u}}$. Furthermore, Schaeffer [58] observed that if the number of individuals per contemporary group is large, treating their effects either as fixed or random is inconsequential. However, the well-known James-Stein theoretical result indicates that shrinkage of fixed effects estimates can yield smaller mean squared of estimation if the number of contemporary groups is large [59]. Hence, Henderson’s prescription is not on solid ground.

Many papers, mainly using simulation, observed that use of “full” relationship matrices in the statistical model could account in some sense for selection, even in situations where missing data lead to non-ignorable selection. In our approach, unless genetic relatedness enters explicitly into the fitness function, there is no transparent theoretical reason for such expectation, other than the benefit stemming from a correct specification of the covariance matrix. As discussed, Henderson et al. [30] observed that ordinary least-squares, assuming independent and identically distributed observations, produced biased estimates of fixed effects under sequential selection, while generalized least-squares did not. In the “gross comparison” of the cow example, all records are used, so selection is based entirely on observed data. However, least squares assumes that first and second lactation records are conditionally independent, inducing a likelihood that is proportional to the product (of densities) $p\left( {\mathbf {y}}_{0}|{\theta}\right) p\left( {\mathbf {y}}_{1}|{\theta}\right) p\left( {\mathbf {y}} _{2}|{\theta}\right)$, so all data used for selection decisions are included but all covariances are ignored. On the other hand, the generalized least-squares estimator derives from a likelihood that is proportional to $p\left( {\mathbf {y}}_{0}|{\theta}\right) p\left( {\mathbf {y}} _{1}|{\mathbf {y}}_{0},{\theta}\right) p\left( {\mathbf {y}}_{2} |{\mathbf {y}}_{1},{\mathbf {y}}_{0},{\theta}\right) .$ Thus, what a genetic relationship matrix does is to represent extant dependencies properly. Apart from an appropriate model specification, the more complete the pedigree (or genomic) information is, the better the dependencies are modelled. The issue here is one of proper specification of the likelihood, i.e., some effects treated as random with an appropriate covariance structure producing shrinkage of least-squares solutions. Henderson et al. [30] referred to this matter as “incomplete repeatability” of records. Ignoring data relevant to the selection decisions does produce distortion in inference, since the fitness function contains information about the unknown ${\theta}$ that is not ignorable.

Harville [60] examined selection using the following setting. Without selection, the data have a distribution with density function $p({\mathbf {y}} |{\theta})$ and with the sample space of ${\mathbf {y}}$ unrestricted in any manner. Selection is such that only data in a restricted space *S* is observed, i.e., when ${\mathbf {y}}{{{\in }}}S.$ Under selection, observations appear with density

92 $$p_{s}\left( {\mathbf {y}}|{\theta}\right) =\frac{p\left( {\mathbf {y}} |{\theta}\right) }{\Pr \left( {\mathbf {y}}{{{\in }}}S|{\theta}\right) };{\mathbf {y}}{{{\in }}}S,$$

so the posterior density under selection, with the prior density being $p\left( {\theta}\right)$, is:

93 $$p_{s}\left( {\theta} {|} {\mathbf{y}}\right) =\frac{\dfrac{1}{\Pr \left( {\mathbf {y}}{{{\in }}}S|{\theta}\right) }p\left( {\mathbf {y}}|{\theta}\right) p\left( {\theta}\right) }{\int \dfrac{1}{\Pr \left( {\mathbf {y}}{{{\in }}}S|{\theta}\right) }p\left( {\mathbf {y}}|{\theta}\right) p\left( {\theta}\right) d{\theta}}\propto \frac{1}{\Pr \left( {\mathbf {y}}{{{\in }}}S|{\theta}\right) }p\left( {{\theta}{|} {\mathbf {y}}}\right) .$$

Since $\Pr \left( {\mathbf {y}}{{{\in }}}S|{\theta}\right)$ depends on ${\theta}{,}$ the selection process cannot be ignored. A well known special case of this type of selection (as noted, also a special case of Pearsonian selection) is the classical truncation model of quantitative genetics. To illustrate this, suppose that, prior to selection, observations are identically and independently distributed as $N\left( \mu ,\sigma ^{2}\right)$ with the variance $\sigma ^{2}$ known but $\mu$ unknown. Selection is such that *n* observations exceeding a known threshold *t* are presented to the analyst and a flat prior is assigned to $\mu .$ The posterior density after selection is:

94 $$p_{s}\left( \mu |{\mathbf {y}},t\right) \propto { \prod \limits _{i=1}^{n}} \frac{\exp \left[ -\dfrac{1}{2\sigma ^{2}}\left( y_{i}-\mu \right) ^{2}\right] }{\Pr \left( y_{i}>t|\mu ,\sigma ^{2}\right) },$$

which is the product of *n* truncated normal density functions. Using standard algebra [25]

95 $$p_{s}\left( \mu |{\mathbf {y}},t\right) \propto \frac{\exp \left[ -\dfrac{n}{2\sigma ^{2}}\left( \mu -y\right) ^{2}\right] }{\left[ 1-\Phi \left( \dfrac{t-\mu }{\sigma }\right) \right] ^{n}},$$

where $\Phi \left( .\right)$ is the normal distribution function. The posterior density of $\mu$ under selection does not have a closed form.

A terminology employed in classification problems, i.e., “hard” versus “soft” [61] may be useful to contrast the treatments of selection employed by [43, 54, 60] with the one we used in the present paper. In “hard selection”, the sampling space *S* implies fixed constraints (e.g., culling levels) defining a simplex, inside of which data are observed. In a “soft selection model”, the fitness or probability of selection depend on arguments that can include fixed hyper-parameters, unknown parameters, observed and unobserved data. The latter corresponds to the missing data treatment examined in [21, 22]. The more realistic and flexible setting in “soft selection” may lead to a diagnosis of the extent to which selection can be ignored. It is not realistic to assume that a fixed selection threshold *t* holds in conceptual replication. The chance of selection depends on varying observed and unobserved data, and on unequal amounts of information over individuals, aspects that the “soft” selection representation addresses.

There does not seem to be a general prescription to accommodate potential distortions due to selection. In structures that combine cross-sectional, longitudinal and multi-trait data such as in animal breeding, balance is the exception rather than the rule. In plant breeding, datasets are more structured and are often outcomes of designed randomized trials. However, such experiments may involve multiple-environments and years and multiple traits. The missing data or fitness treatment presented here may be also pertinent to data from designed experiments, where missing observations also occur in incomplete block layouts, and the missingness may not be random. The Bayesian approach, together with our treatment of selection, offer an integrated answer to inference, prediction and model selection [25, 62, 63] and goes beyond the likelihood-based approach, where breeding values are inferred indirectly. In the Bayesian treatment, the fitness function can include data, parameters and breeding values, as the latter are members of the vector of unknowns, although assigned distinct prior distributions. Bayesian methods produce automatic measures of uncertainty even under selection and the posterior distribution of the fitness function can be estimated using draws from its posterior distribution.

Is it always fruitful to account for selection by introducing a fitness function or by modeling the missing data process? Modeling selection through a fitness function is not without pitfalls. An incorrect specification of fitness may deteriorate inferences beyond those obtained by ignoring selection altogether. Accounting for selection may not reduce uncertainty about breeding values, as we found in our examples with real data, where the missing data process assumed, univariate or multivariate, patterned exactly the protocols constructed. More generally, since the quality of inferences cannot be assessed unambiguously (one does not know the “true value” of parameters), it is risky to assert that inferences are good or bad. Formal model comparison may shed some light. For instance, a Bayes factor analysis may reveal that accounting for selection provides a more plausible description of the data than ignoring selection.

Lastly, since animal and plant breeders are interested in predicting future phenotypes, a predictive assessment may be the most appropriate gauge for constructing and calibrating models representing competing forms of describing the selection process. For example, Gianola and Schön [64] address several ways of carrying-out cross-validation, directly or indirectly. From a Bayesian perspective, Fong and Holmes [65] argue that the marginal density of the data (denominator of Bayes theorem) is “equivalent” to exhaustive leave-*k* out cross-validation averaged all possible values of *k* when a log-posterior predictive distribution is used as scoring rule for competing models. However, their theoretical results depend on the notion of “exchangeability”, i.e., that permutation of indexes of observations does not alter the analysis. This concept does not apply to quantitative genetics settings, since, for example, if a parent is individual *i*,  say, the analysis would change drastically if it is permuted with grand-children *j*. Another example from dairy cattle breeding is as follows: if a cow is *m*,  its production record cannot be exchanged with bull *n*,  with thousands of progeny. Obviously, a bull cannot be milked and a cow can seldom produce such a large progeny group of individuals.

## Conclusions

We reviewed and extended theory for analyzing quantitative genomics data stemming from cryptic or structured selection processes. The Bayesian approach provided an integrated approach to inference and prediction under selection, but may or may not yield the best possible predictions, as each problem is essentially unique. One may believe that selection has been accounted for meticulously, but the central question of whether inferences are good or bad does not have an answer. As pointed out by Mark Twain: “*It ain’t what you don’t know that gets you into trouble. It’s what you know for sure that just ain’t so*”. It may well be that some statistical learning procedure that ignores quantitative genetics theory and non-randomness ends up as the best prediction machine. Since the most celebrated prediction and classification machines do not make claims about lack of bias or minimum variance of structural parameters, e.g., connection strengths of deep neural networks, these two concepts largely driving the Hendersonian era (at least in animal breeding) may gradually lose relevance. On the one hand, Bayesians may experience a certain *schadenfreude*^1^ if this were to occur. On the other hand, it is possible to attain empirically unbiased predictions via calibration of the machines. A positive finding may not help to understand the state of nature, but it may enhance the progress of agriculture.

## Data Availability

The wheat data set is publicly available.

## Footnotes

1 A certain pleasure derived by someone from another person’s misfortune.
